# New Class of Polymer Materials—Quasi-Nematic Colloidal Particle Self-Assemblies: The Case of Assemblies of Prolate Spheroidal Poly(Styrene/Polyglycidol) Particles

**DOI:** 10.3390/polym14224859

**Published:** 2022-11-11

**Authors:** Damian Mickiewicz, Mariusz Gadzinowski, Tomasz Makowski, Witold Szymański, Stanislaw Slomkowski, Teresa Basinska

**Affiliations:** 1 Division of Functional Polymers and Polymer Materials, Centre of Molecular and Macromolecular Studies, Polish Academy of Sciences, H. Sienkiewicza 112, 90-363 Lodz, Poland; 2 Institute of Materials Science and Engineering, Lodz University of Technology, B. Stefanowskiego 1/15, 90-924 Lodz, Poland

**Keywords:** polymer prolate spheroidal particles, poly(styrene/polyglycidol) particles, polymer microspheres, colloidal crystals, self-assembly of particles, nematic arrangement, particles array, nanoindentation, mechanical properties of particles arrays

## Abstract

Assemblies of colloidal polymer particles find various applications in many advanced technologies. However, for every type of application, assemblies with properly tailored properties are needed. Until now, attention has been concentrated on the assemblies composed of spherical particles arranged into so-called perfect colloidal crystals and on complex materials containing mixtures of crystal and disordered phases. However, new opportunities are opened by using assemblies of spheroidal particles. In such assemblies, the particles, in addition to the three positional have three angular degrees of freedom. Here, the preparation of 3D assemblies of reference microspheres and prolate spheroidal poly(styrene/polyglycidol) microparticles by deposition from water and water/ethanol media on silicon substrates is reported. The particles have the same polystyrene/polyglycidol composition and the same volumes but differ with respect to their aspect ratio (*AR*) ranged from 1 to 8.5. SEM microphotographs reveal that particles in the assembly top layers are arranged into the quasi-nematic structures and that the quality of their orientation in the same direction increase with increasing *AR*. Nano- and microindentation studies demonstrate that interactions of sharp and flat tips with arrays of spheroidal particles lead to different types of particle deformations.

## 1. Introduction

According to the Web of Science Database from the beginning of the XXI century, the number of yearly published papers concerning colloidal crystals increases continuously. Interest in particle arrays is due to the growth of applications of these materials in many advanced technologies. Colloidal crystals were used as elements of miniaturized spectrometers [1], optical and chemical sensors [2,3,4,5], optical data recorders [6], light guides [7], and many other appliances. In spite of still-unresolved problems of preparation of materials with complete photonic band gap, the particles assemblies arranged in crystalline order are in demand.

Colloidal object arrays can be formed as a result of self-assembling constituent particles under action of entropy and/or enthalpy driven forces [8]. Some methods are based on controlled action of applied external stimuli. Among them, procedures enabling fabrication of regular colloidal crystals are extremely useful. They include vertical deposition [9,10], gravitational sedimentation [11], membrane filtration [12], emulsion crystallization [13], or Langmuir–Blodgett-based methods [14,15].

Recently, the composites of carboxylated polystyrene particles and micelles were formed involving supramolecular bonds between carboxyl groups of particles and poly(ethylene oxide) block of copolymer micelles. The arrangement of the composite material and supramolecular crosslinking was controlled kinetically by a pH change of the environment [16]. 

The disadvantage of using spherical particles in colloidal photonic crystals is not the complete band gap resulting from symmetry induced degeneracy of the lattice [17]. One of the possible solutions consists in replacement of spherical by spheroidal particles in the unit cell, yielding materials with complete band gap [18]. Moreover, prolate spheroidal particles attract attention as potential drug carriers, as their surface is larger than the surface of spheres with the same volume [19]. There are some hopes that particles with a larger surface would more efficiently react with biomolecules and even with whole living cells. However, there are some problems with large-scale application of microspheroids for fabrication of crystalline colloidal assemblies. Namely, existing methods useful for fabrication of spheroidal particles on a laboratory scale are difficult to scale-up. Moreover, the formation of arrays of spheroidal particles is much more complex than in the case of arrays of spheres and, in spite of some progress, still requires intensive investigations [20]. 

There are reports that the well-oriented arrays were obtained as a result of spheroidal particle assembly under action electric or magnetic field and by using lithography methods. For instance, as a result of combining of self-assembling of prolate particles and alternating electric fields, which induced dipolar interactions, the reversible formation of regular virus-like (tubular) structures was observed [21]. Very dense and ordered assemblies were produced by electrophoretic deposition and constant electric field-assisted assembly [22]. Similarly, fully organized colloidal crystals were obtained from highly charged silica rod-like particles by deposition in constant electric field [23]. The orientation of particles in these crystals could be switched by changing direction of electric field. In other work, the external magnetic field was used for the preparation of 3D hematite photonic crystals [24]. Hematite spindles used in the aforementioned studies were coated with silica shells, yielding spheroidal particles with an aspect ratio of 1.3 and 1.5. The microspheroids in the crystals revealed good positional and orientational order.

Lithography was used to manufacture alumina rods arrays for waveguide devices in the gigahertz range [25]. The assemble-and-stretch-and-combine procedure for preparation multilayer particle arrays was also elaborated on, with morphology controlled at each particle layer [26]. The process consisted of assembling spherical uniform particles into 2D (one microsphere thick) colloidal crystal, subsequently embedding the crystal into a poly(vinyl alcohol) (PVA) film, stretching the film and preparation of multiple (layer by layer) film stacks. Finally, the 3D crystals with well-controlled arrangement of spheroidal particles by film dissolution were produced.

Arrays of microparticles were also formed by a simple microparticle deposition in the gravitational or centrifugal field [27,28]. However, relations between particle shape and arrays morphology are not well understood and require further study.

The construction of many devices used in bioscience requires materials with properly tailored hierarchical structure and surface properties. This includes sensors detecting various biomolecules, simple ions, measuring pH, and providing information on biochemical processes that take place in various parts of the human body. Often, hydrophilic materials with controlled surface morphology are needed; materials equipped with specific chemical groups enabling binding of biomacromolecules (e.g., proteins or nucleic acids), cellular organelles, or even whole cells. Arrays of colloidal particles are interesting candidates for the aforementioned applications. However, not all properties of these structures were sufficiently well-investigated. The optical properties of particles arrays are already quite well known, while their mechanical properties are not. The modulus of elasticity and hardness could play an important role in case of materials used for construction of detectors in biosensors. For obvious reasons, they should be sufficiently robust. However, often this is not the case. For example, the polystyrene colloidal crystals made from microspheres are very friable, difficult to manipulate after drying, and require additional chemical modification. Moreover, elasticity of adsorbing surface affects adsorption of living cells [29].

It is known that spheroidal particles could be more densely packed than spherical ones [30]. Using simulation, it was found that typical crystal arrangement for spheres in cells contains six particles, forming a hexagon. However, stable packing of spheroids requires ten particles. Thus, materials composed from spheroids should be much stronger than their equivalents from spherical particles [30]. The simulation studies of random packing of spheres and spheroids revealed that volume fraction (φ) of spheroids equal 0.74 is much higher than volume fraction of packed spheres, for which φ was 0.64 [31]. It was suggested that the higher density of the spheroid arrays than the density of the arrays of spheres is directly related to their higher number of degrees of freedom per particle [30]. 

Arrays of spheroidal particles were usually produced under the action of controlled external stimuli. The aim of this work was investigation of spheroidal particle 3D arrays formed by the simplest process of drying suspension of particles on silicon supports in the field of natural gravity. The goal was to find out relations between particle aspect ratio (*AR*, ratio of long and short particle axis) and quality of particle order in the arrays. The studies included determination of particle orientation in interfacial layer of particles assemblies and some information of particle arrangement in bulk. These studies could be compared with the earlier work on 2D (one particle thick) arrays of microspheroids [32]. The research was also directed on angle-resolved UV light diffraction from the arrays formed by the aforementioned method. Although mechanical studies were performed for polymeric colloidal supra-assemblies, to our best knowledge, the paper presents results of studies of elasticity and hardness of the arrays of polymer microspheroids for the first time [33,34]. The measurements of nano- and microindentation were performed using sharp Berkovich and flat-punch tips. The small roundness (r ≤ 20 nm) of the Berkovich tip enabled determination of local mechanical properties (modulus of elasticity and hardness) of the array, whereas testing using the flat-punch tip (usually with diameter in a range from 20 to 200 μm) provided information on the modulus of elasticity of the much larger part of the surface of the array.

Particles with polystyrene cores and polyglycidol-rich shells were used in this work. The polystyrene core provided robustness of the particles and polyglycidol-rich shells—their hydrophilicity and functionality (due to the presence of –CH_2_OH groups). Particles with *AR* ranging from 2.17 to 8.50 and the pristine microspheres with diameter *D*_n_ = 408 nm were used for the studies. Spheroids were produced by: (i) embedding the pristine microspheres in poly(vinyl alcohol) (PVA) film, (ii) uniaxial film stretching for required time at temperature above *T*_g_ of polystyrene and polyglycidol, and (iii) purification from unbound PVA by a sequence of multiple centrifugation/redispersion in pure water. The details of the aforementioned method were described in some earlier papers [35,36]. The pristine microspheres and spheroids were characterized by size, surface charge, and interfacial layer composition.

## 2. Materials and Methods

### 2.1. Materials

Styrene, glycidol, ethyl vinyl ether, *p*-toluene sulfonic acid, *p*-chloromethylstyrene, Hyflo Super gel (Aldrich), aluminum chloride (AlCl_3_ × 6H_2_O, Sigma-Aldrich, St. Louis, MO, USA), poly(vinyl alcohol) (PVA) with *M*_w_ in the range 31,000–50,000 (87–89% hydrolyzed, Sigma-Aldrich), sodium chloride (Sigma-Aldrich), sodium bicarbonate (Sigma-Aldrich), 5-([4,6-Dichlorotriazin-2-yl]amino)fluorescein hydrochloride (Sigma-Aldrich), silicon wafers (single side polished, N+/Sb <100>, coating 306 nm, Si-MAT, Silicon Materials, Glenshaw, PA, USA), glass plates for optical microscopy (Thermo-Scientific, Waltham, MA, USA), methylene chloride (POCh), dimethylformamide (Chempur), isopropanol (POCh), ethanol (96%, freshly distilled, POCh), and deionized water obtained from a water purification system (ADRONA, Riga, Latvia).

### 2.2. Methods

The synthesis of α-tert-butoxy-ω-vinylbenzyl-polyglycidol macromonomer and poly(styrene/polyglycidol) (P(S/PGL)) microspheres is described in Methods S1.

The procedure for preparation of P(S/PGL) spheroidal particles from the P(S/PGL) microspheres is described in Methods S1.

The procedure for hydrophilization of silicon and glass plates for studies of particle deposition is described in Methods S1.

#### 2.2.1. Preparation of Suspensions of P(S/PGL) Spheroids for Deposition on Glass/Silicon Supports

The multilayers of the spherical or spheroidal particles prepared from their suspensions in water or water/ethanol mixtures were produced as follows: the sample of particles underwent centrifugation and aqueous supernatant was replaced with water/ethanol mixture with various ethanol concentration (10, 20, and 30 vol%). Next, the particles were redispersed in mixed solvents using a minishaker (IKA, Staufen, Germany). The volume of water/ethanol mixture in each sample was adjusted to get desirable particles concentration (equal 100 mg/mL). The water or water/ethanol suspension of particles ((*c* = 0.1 g/mL, *v* = 50 µL) was deposited on dried silicon wafers and left to dry at ambient atmosphere. The surface area of the formed particle multilayer was ca. 2 cm^2^ and their thickness varied from 40 to 80 μm for various samples.

Analytical methods used in the studies of particles preparation and arrays, such as ^1^H NMR, XPS, AFM, SEM, surface tension measurements, determination of zeta potentials of spherical and spheroidal particles, and nanoindentation measurements are described in Analytical Methods S2.

#### 2.2.2. Determination of PVA Traces Irreversibly Adsorbed on the Surface of Spherical and Spheroidal Particles

Irreversible adsorption of PVA on the particle surface was monitored using PVA labeled with fluorescent labels. Labeling was performed using 5-([4,6-dichlorotriazin-2-yl]amino)fluorescein hydrochloride (5-DTAF), according to the method described in ref. [37]. Briefly, the procedure of labeling was as follows: sample of PVA (60 mg) was dissolved in carbonate buffer (10 mL, I = 0.1 M, pH = 9.3) and subsequently added to 5-DTAF (31 mg) dissolved in DMSO (2 mL). The PVA/5-DTAF ratio was chosen in a way enabling functionalization of 0.05 mol% PVA hydroxyl groups fraction. The functionalization was carried out for 4 h at ambient temp. in the dark. Then, the mixture was dialyzed against di. water. Dialysis was completed when absorbance spectrum of water in dialysis container was free from signal of 5-DTAF (maximum at *λ* = 535 nm). 

The determination of 5-DTAF-labeled PVA adsorbed on particles during contact with PVA film was performed in the following way. The sample containing 5-DTAF (15 mg) labeled PVA and unlabeled PVA (0.95 g) was dissolved in di. water (7.0 mL) at 50 °C. Then, aqueous suspension of microspheres (0.7 mL, conc. 0.1 g/mL) was added to the PVA aqueous solution. The mixture was deposited on a Petri dish and water was evaporated at ambient temperature. The produced film with embedded microspheres was dissolved in di. water (90 mL) and the mixture with suspended microspheres was centrifuged (using 380R, MPW Med. Instruments, Poland) to separate the supernatant. The resuspension/centrifugation cycle was repeated several times until the supernatant was 5-DTAF free. The 5-DTAF content in each collected supernatant sample was determined by UV-VIS absorption spectroscopy (Specord S600, Analytic Jena, Jena, Germany). Finally, the sample of particles was lyophilized in vacuum. Thereafter, lyophilized particles (40 mg) were dissolved in *N,N*-dimethylformamide (1 mL, DMF) containing NaHCO_3_ (20 mg). The dissolution process was carried out for 24 h at ambient temp. Thereafter, the absorption spectrum was registered and PVA content was determined using the previously prepared calibration curve of 5-DTAF in DMF/NaHCO_3_. The determination of PVA adsorbed on the surface of spheroidal particles was very similar to the aforementioned procedure for determination of PVA on spherical particles. The PVA film with embedded P(S/PGL) microspheres was elongated (at 120 °C) and the produced spheroids were isolated from the film using washing of particles by repeated resuspension/centrifugation. For preparation of film of PVA (1.996 g), PVA-5-DTAF (56 mg) was dissolved in di. water (14 mL). Further steps yielding spheroidal particles with adsorbed 5-DTAF labeled PVA were identical to the procedure described for separation of regular spheroids from PVA (see preparation of poly(styrene/α-tert-butoxy-ω-vinylbenzylpolyglycidol) (P(S/PGL) spheroidal particles described in Methods S1).

#### 2.2.3. Recording of UV-Vis Angle-Resolved Reflectance Spectra

The angle-resolved diffraction spectra of deposited colloidal assemblies were recorded using a variable angle reflectance attachment fixed in the UV-VIS spectrophotometer (Specord S600, Analytic Jena, Germany). The aforementioned attachment enabled registration reflectance spectra at light incident angles in the range 10–60° and in the range of wavelengths 220–900 nm. For all analyzed samples of colloidal arrays, the reflectance spectra were recorded at angles *θ* ≥ 45°. The wavelengths at maxima of reflectance intensity were registered and used for preparation of plots of *λ_max_*^2^ (maximum of reflection) as function of *sin*^2^*θ*, where *θ* denotes light incidence angle. From the dependence of *λ*^2^ versus *sin*^2^*θ*, the distance between diffracting planes was determined.

#### 2.2.4. Measurements of Water Contact Angle Deposited on Colloidal Multilayers

The water contact angle was assessed after deposition of 2 µL di. water on freshly prepared assemblies of spherical and spheroidal particles (on each of two assemblies prepared independently). The measurements were performed using Phoenix-300 goniometer (SEO Surface Electro Optics, Suwon-si, Korea). The registered contact angle was an average of the three independent contact angles measured immediately after the deposition of a drop of water.

#### 2.2.5. Measurements of Mechanical Properties by Nanoindentation Method

The nanoindentation measurements were performed using an MTS Nano Instruments model G 200 Nano Indenter (Agilent Technologies, Santa Clara, CA, USA) equipped with an antivibration table, antivibration chamber with an acoustic/thermal isolation, CSM controller, and NanoSwift controller. The diamond flat punch tip cylindrical penetrator had a diameter of 100 μm and a diamond Berkovich tip with a semi-angle of 65.3° and tip roundness of r ≤ 20 nm (both from Micro Star Technologies, Huntsville, TX, USA). Samples were mounted in the following way. First, the glass plate was attached to the holder (aluminum sample disk) by a standard procedure based on using a crystal bond binder (melting temperature approximately 135 °C). Then, after cooling, a silicon plate with deposited array of particles was glued to the glass plate with the cyanoacrylate glue. The quasi-static test with the flat punch tip penetrator was carried out using the 0.4 mN load. The loading and unloading time was 1 s and the time of maintaining the sample at maximum load was 5 s. The loading/unloading curves were recorded and used for determination of the reduced modulus of elasticity (*E_r_*). For the flat point end nanoindenter with a circular cross-section of its cylindrical part, *E_r_* could be calculated using Equation (1):

(1) $$E_{r}=\frac{S}{D}$$

where *S* is the slope of the upper part of the unloading curve and *D* is the diameter of the nanoindenter [38].

Equation (2) describes the relation between the measured reduced modulus of elasticity (*E_r_*) and modulus of the investigated sample (*E_S_*):

(2) $$\frac{1}{E_{r}}\;=\frac{\left(1-\nu_{u}^{2}\right)}{E_{S}}+\frac{\left(1-\nu_{tip}^{2}\right)}{E_{tip}}$$

where *ν_u_* = 0.25 denotes the universal value of Poisson’s ratio used for polymer materials (for pure polystyrene *ν_polystyrene_* is slightly higher and equals 0.33), and *ν_tip_* and *E_tip_* denote Poisson’s ratio and modulus of elasticity of diamond tip, respectively.

It is worth noting that for diamonds, the modulus of elasticity is very high (1143 GPa) and Poisson’s ratio quite low (0.0691) [39]. With these parameters, the second term in the right-hand side of Equation (2) is about 10^8^ times smaller than the first and can be neglected. As a result, Equation (2) can be simplified and rewritten as:

(3) $$E_{S}=0.9375\;E_{r}$$

The modulus of elasticity and hardness was determined using a sharp tip (Berkovich) and 1 mN loading. The time regime of applying forces was the same as in the case of the tests with flat punch nanoindenter, i.e., 1 s for loading/unloading and 5 s for maintaining sample at maximum load. For tests with Berkovich nanoindenter, the equipment automatically provides values of modulus of elasticity and hardness.

## 3. Results and Discussion

### 3.1. Preparation and Basic Characteristics of Spherical and Spheroidal P(S/PGL) Particles

Prolate spheroidal particles were prepared from microspheres with a polystyrene core and a polyglycidol-enriched shell. The synthesis of microspheres and preparation of spheroidal particles are described in Methods S1. The number average diameter of microspheres (*D*_n_) was 408 nm (measured from SEM microphotographs) and the dispersity factor *D*_w_/*D*_n_ = 1.04 indicated that very uniform particles were obtained. It is worth noting that the diameter of particles determined by PCS (Photon Correlation Spectroscopy) was equal to 430 nm, slightly larger than the diameter found by analysis of SEM microphotographs. The 22 nm (11 nm of the radius) difference indicated that the particle interfacial layers were swollen in water due to the presence of polyglycidol segments [40]. The molar fraction of polyglycidol in the particle’s interfacial layer, available for XPS analysis (ca. 7 nm thick), was 0.216. The particles were negatively charged due to sulfate anion end-groups in polystyrene chains formed during initiation with K_2_S_2_O_8_ [41]. 

The elongation of the PVA film with embedded P(S/PGL) microspheres at 120 °C (i.e., slightly above *T_g_* of particles and PVA) yielded prolate spheroids [36]. It should be noted that the elongation degree of microspheres (*α*) is proportional to the elongation degree of the film (see Ref. [36] and Figure S4), and that the aspect ratio (*AR*) of these particles is related to their elongation degree as $AR=\alpha \sqrt{\alpha }$. In this work, spheroids with *AR* ranging from 2.17 to 8.50 were used (see Table 1).

#### Chemical Composition of (P/S/PGL) Microspheres and Microspheroids. Determination of Adsorbed PVA on the Particle Surface

It was reasonable to expect that the process of spheroid formation, which was used in this work, could be accompanied by changes in the chemical composition of the particle interfacial layer. These changes may have the following reasons: some rearrangement of polymer chains resulting from particle stretching and/or simply irreversible adsorption of PVA during contact of particles at 120 °C with the PVA matrix. The role of PVA adsorption was assessed from analysis of XPS spectra of the pristine P(S/PGL) microspheres (in Table 2 the sample denoted as P(S/PGL)_m_) and microspheres, which, embedded in PVA film were conditioned at 120 °C and thereafter isolated by dissolution of PVA film matrix in water and purified by at least 20 repeated cycles of centrifugation and resuspension in fresh portions of water (sample P(S/PGL)_m_-PVA). Also investigated were spheroids (sample P(S/PGL)_s_3). Deconvolution of the high resolution of the XPS C1s signal (Figure S5) of P(S/PGL)_m_ revealed the presence of the following groups: CC aromatic (284.75 eV, phenyl groups of polystyrene, and benzyl groups of polystyrene units *p*-substituted with polyglycidol), CC aliphatic groups (284.99 eV; in polystyrene and polyglycidol main chains), ether COC groups (286.13 eV present in polyglycidol main chain), and alcohol CH_2_OH groups (286.67; present in polyglycidol side groups). In addition, the spectrum contained the shake-up satellite at 291.4 eV from phenyl ring of polystyrene and benzyl groups of polyglycidol. The XPS spectra of particles (microspheres P(S/PGL)_m_-PVA and spheroids P(S/PGL)_s_3 isolated from PVA film were different from the XPS spectrum of the pristine microspheres (see Figure S5). First, in the spectra of particles, which for some time were embedded in PVA film, the COC ether signal was absent and the signal of C-C aromatic moieties was weaker. Moreover, the signal of aliphatic C-C groups was much higher for microspheres and microspheroids, the surfaces of which were in contact with PVA film, than for the pristine microspheres. The aforementioned observations indicate that at 120 °C contact of particles with PVA leads to their coating with adsorbed PVA chains, which cannot be desorbed even after multiple (repeated up to 20 times) washings with fresh portions of water. This conclusion was supported by results of zeta potential measurements of P(S/PGL) microspheres and microspheroids (see Figure S6). All of them have negative charge because the microspheroids were obtained from microspheres that were synthesized and purified in a process yielding polymers with -SO_4_^−^H^+^ end-groups. Dissociation of protons gave particles with anions -SO_4_^−^ and thus with negative zeta potential −30.8 mV. The addition of NaCl increasing concentration of Na^+^ cations resulted in replacement of protons with sodium cations, which dissociate easier making zeta potential of microspheres more negative (−56.2 mV, for concentration of NaCl 5 × 10^−3^ M/L). A further increase of NaCl concentration increased the number of sodium ions in the ionic double layer at the surface of microspheres, making their zeta potential less negative (−48.4 mV for concentration of NaCl equal 10^−2^ M/L). Coating microspheres and microspheroids with irreversibly attached PVA adlayer during their processing in PVA films should bury at least some ionic species, making the zeta potential of such particles less negative. This effect can be seen in Figure S6. 

In order to determine how large an amount of PVA remained sustainably adsorbed on the particle surface, the fraction of PVA labeled with 5-([4,6-dichlorotriazin-2-yl]amino)fluorescein was used for the preparation of spheroids. The determination procedure is described in Section 2.2.2. The spherical particles were used as a reference sample for comparison to the result obtained for PVA fraction absorbed on the P(S/PGL) spheroid surface. The results presented in Table 2 revealed that PVA wt% fraction adsorbed on the surface of microspheres and spheroids was 5.0 and 5.8 wt%, respectively.

It is worth noting that the fraction of PVA (expressed as %(wt/wt)) adsorbed onto the microspheroids was slightly larger than the fraction of PVA adsorbed onto the pristine P(S/PGL) microspheres. This might be attributed to the fact that deformation of the original spherical particles increases their surface.

Remembering that the volume of (P(S/PGL) microparticles does not change during stretching [36] and that volumes of microspheres (*V*_m_) and microspheroids (*V*_s_) are described by the following formulae: (4/3)*πr*^3^ and (4/3)*πa*^2^*c*, respectively (where *r*, *a*, and *c* denote radius of microsphere and short and long semi-axis of prolate microspheroid), the following relation holds (Equation (4)):

(4) $$r^{3}=a^{3}AR,\;where\;AR=\frac{c}{a}$$

For microspheres with *r* = 204 nm and *AR* = 6.41 (radius of P(S/PGL)_m_-PVA and aspect ratio of P(S/PGL)_s_3 microparticles, respectively) *a* = 109 nm and *c* = 698 nm. Surfaces of the P(S/PGL)_m_ and P(S/PGL)_s_3 microparticles (denoted as *S*_m_ and *S*_s_, respectively) were calculated using formulae shown below (Equations (5) and (6)):

(5) $$S_{m}=4\pi r^{2}$$

(6) $$S_{s}=2\pi a\left[a+\frac{c^{2}}{\sqrt{c^{2}-a^{2}}}\;arcsin\left(\frac{\sqrt{c^{2}-a^{2}}}{c}\right)\right]$$

Therefore, for P(S/PGL)_m_ *S*_m_ = 5.23 × 10^5^ nm^2^ and for P(S/PGL)_s_3 *S*_s_ = 7.59 × 10^5^ nm^2^ indicate that surface of P(S/PGL)_s_3 microspheroids is 45.1% larger than surface of corresponding P(S/PGL)_m_-PVA microspheres. Because the amount of PVA irreversibly attached onto P(S/PGL)_s_3 is only by 16% larger than the amount of PVA adsorbed on microspheres (P(S/PGL)_m_); see Table 2, it would be reasonable to assume that PVA is not evenly distributed on the surface of microspheroids but forms domains, probably due to interactions with polyglycidol rich domains. It is worth noting that domain morphology of surfaces of P(S/PGL) microspheres and microspheroids was already noticed in our earlier studies [36].

### 3.2. Properties of Multilayers of Microspheroids

The deposition of P(S/PGL) microspheroids aqueous and water/ethanol suspensions (with particles conc. 100 mg/mL) on silicon supports yielded 3D multilayers. The particle arrangements occupied ca. 2 cm^2^ of the plates.

**Figure 1:**
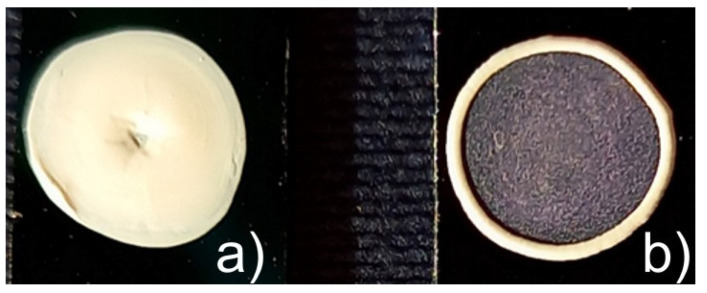
Digital photos of drops of aqueous suspensions of (**a**) spheroidal particles (P(S/PGL)_s_3 and (**b**) microspheres P(S/PGL)_m_ after deposition on silicon supports and drying at room temperature.

Microspheroids with an aspect ratio in the range of 1.0–8.5 self-assembled into uniform stains. Moreover, contrary to the deposits of microspheres, the assemblies of microspheroids were free from a boundary of so-called “coffee stain”, as is visible in Figure 1, and showed good adhesive properties to the hydrophilic support. The absence of “coffee-ring” for assemblies of microspheroids resulted from interface deformation during particle deposition and evaporation of the liquid. The liquid is pulled up near the middle of the microspheroidal particle, and simultaneously is pushed down near its tips. In effect, the long-range lateral capillary forces affect the arrangement of microspheroids at the interface [42]. Thus, the microspheroidal particles are uniformly distributed at the air/liquid interface regardless of the particle aspect ratio, providing it is higher than 1 [42]. In consequence, the local density of microspheroids in deposited assemblies is uniform with various particle arrangements.

Furthermore, the composition of spheroids surface layer enriched in hydroxyl groups (derived from PVA and polyglycidol segments) promotes hydrogen bond interactions between particles and accelerates their self-assembling. Moreover, the water-swollen shells enhanced particle self-assembling due to their viscoelastic deformation. However, the addition of ethanol to the aqueous suspension can decrease inter-particle hydrogen bonds. In turn, the capillary forces would dominate in the formation of final particle arrangements [43].

#### 3.2.1. AFM Studies of Particle Interfacial Layers and Particle Assemblies

**Figure 2:**
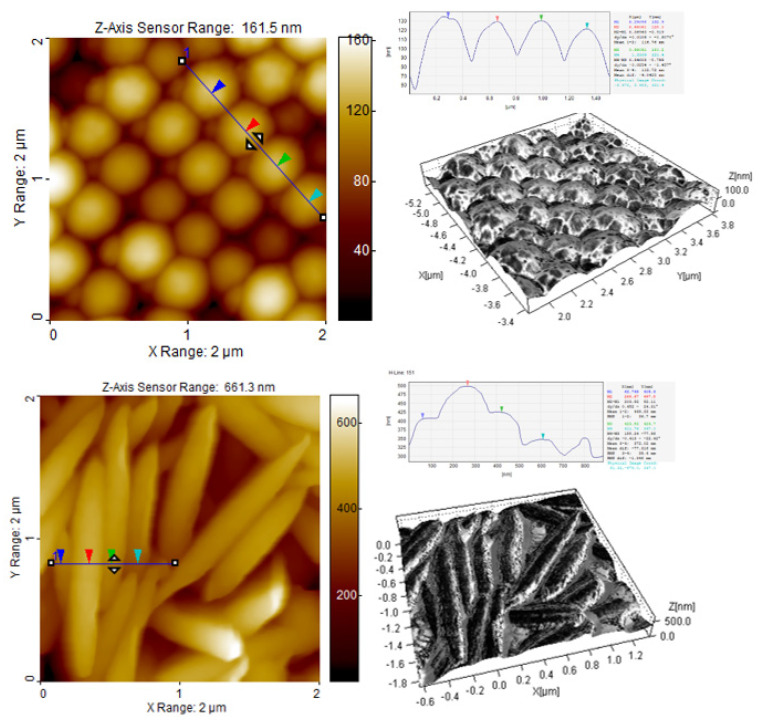
AFM height images of spherical and spheroidal (*AR* = 6.41) particle multilayers and 3D AFM phase contrast images of P(S/PGL)_m_ and P(S/PGL)_s_3 particles deposited from aqueous suspensions.

The topology of the surface of the particle multilayer was investigated by AFM. Figure 2 presents AFM images of 2 µm^2^ of multilayers of P(S/PGL) microspheres and microspheroids. The height images display the morphology of the surface composed of spherical and spheroidal particles. It is worth noting that the spherical particles are in very close contact with each other and a form crystalline arrangement with particle tops at the same height on the whole analyzed area. The distance from top of one particle to top of adjacent particle was 370 nm. However, for the spheroidal particles, a different picture is observed. Namely, the spheroids were placed in some distance one from another and they observed considerably larger irregularities in surface morphology. It is evident that the differences of heights in an analyzed area result from non-uniform packing of deeper located particles with interlocked tips.

The average length of short axis of P(S/PGL)_s_3 particles was 202 nm, i.e., is very close to the average length of short axis determined by SEM (204 nm).

It is worth stressing that surface irregularities are on the nano- and micrometer size. Thus, taking into consideration the application of multilayers of microspheroids for biomedical purposes, it should be taken into account that surface irregularities may affect interactions of proteins or cells with objects made from these materials.

The 3D AFM phase contrast images revealed that surfaces of particles were not isotropic with respect to their viscoelastic properties. It has already been noticed that the stretching of microspheres resulted in the rearrangement of nanodomains on their surface. The randomly distributed nanodomains on surfaces of the P(S/PGL) microspheres were transformed into stripes arranged perpendicularly to the elongation axis [36].

It is worth noting that hydrophilicity of the arrays of the P(S/PGL)_m_ microspheres and P(S/PGL) microspheroids strongly depends on the time of their contact with water. Immediately after deposition of a drop of water on the surface of particle array (regardless of whether they were spherical or spheroidal with any aspect ratio), the contact angle was in the range 52–57° (see data in Table S3). However, a few seconds later, the drop sank into the particle multilayer. Rapid water penetration into particle assemblies, at least having the partially random rough surface, suggests the contribution of hydrophilic polyglycidol-PVA particle shells in the process of soaking water into this kind of material. Rapid hydrophilization of P(S/PGL) assemblies can be advantageous for covalent immobilization of bioactive molecules and reduction of adventitious adsorption of proteins.

#### 3.2.2. SEM Studies of Particle Assemblies

Reports on detailed studies of forces acting on spheroidal particles trapped in fluid interfaces and on capillary forces responsible for interactions between two spheroidal particles located in the fluid interfacial region have been already published [44]. There were also capillary interactions investigated between microspheroidal particles at water–oil [45] and water–air interfaces [46]. However, there was a lack of quantitative studies of morphology of solid multilayers of microspheroids formed by a simple drying process.

**Figure 3:**
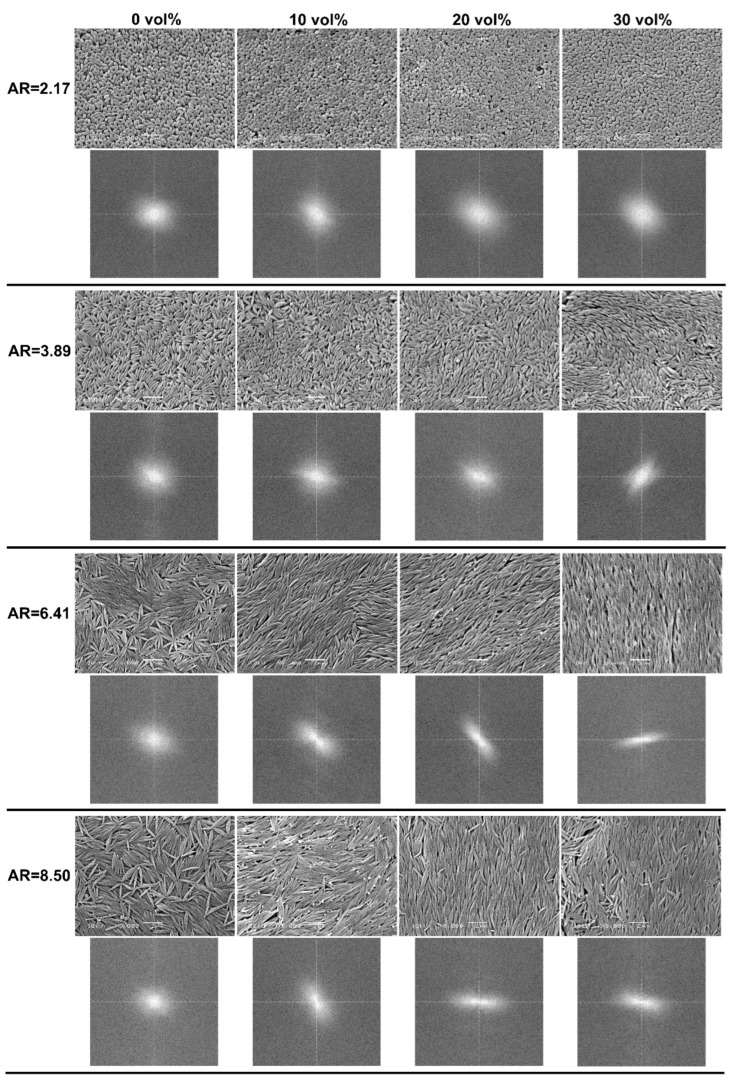
Representative SEM images of top layer of multilayered assemblies of P(S/PGL) microspheroids with various aspect ratios (*AR*) and concentration (0–30%) of ethanol in aqueous particle suspension and corresponding two-dimensional fast Fourier transformation (FFT) images.

Figure 3 presents SEM microphotographs showing the top view of representative arrangements of P(S/PGL) microspheroids. Below each image, the corresponding two-dimensional 2D fast Fourier transform (FFT) is present. It should be noted that microspheroids at the surface of particle assemblies to some degree maintain the angular order, and that the quality of this order depends on the ethanol content in the liquid phase of microspheroids suspensions and on the particle aspect ratio. Visual inspection suggests that better cooperative orientation was achieved for a higher content of ethanol, however, especially strong improvement was noticed when microspheroids with higher values of *AR* were used. The FFT images confirmed the ordering of microspheroids in the top layers of particles. 

It is difficult to explain the changes in arrangement of microspheroids in relation to increasing the ratio of ethanol in particle suspensions. Nevertheless, it is clear that water plays an important role in swelling the polyglycidol-rich interfacial particle layer. On the basis of results of our previous studies on photonic colloidal crystals prepared in water from the polystyrene core/polyglycidol-rich shell microspheres, the interfacial layer forms interparticle bridges [47]. Similar interactions may also exist between microspheroids with similar core-shell morphology. Increasing ethanol content in water/ethanol continuous phase may decrease swelling of particle interfacial layer, reduce particles “gluing”, and enable their easier reorientation and better fitting to the already existing assembly.

It is obvious that quantitative characterization of the arrangement of microspheroidal particles in surface layer and bulk of particle assemblies would be helpful for proper comparison of their morphology. The subsequent part of the discussion will be devoted to this subject.

The arrangements of particles, shown on several microphotographs in Figure 3, resemble colloidal nematic liquid crystals. Thus, in this work, the analysis will be limited only to this type of structure.

**Figure 4:**
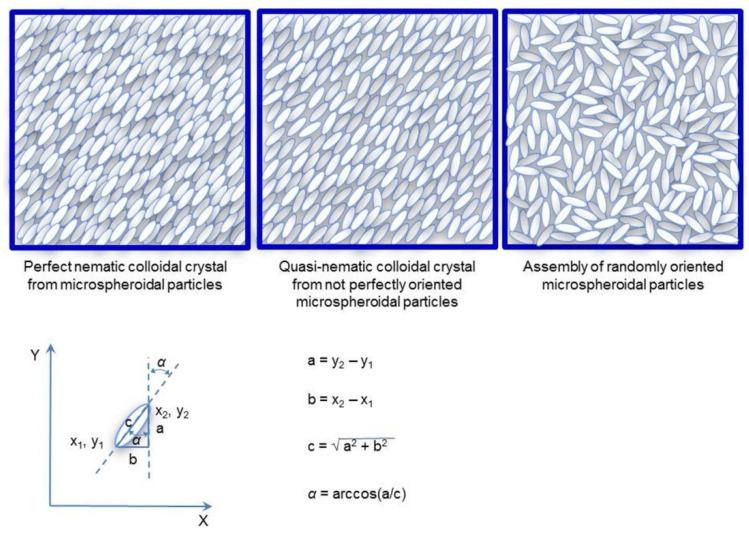
Schematic illustration of perfect nematic, quasi-nematic colloidal crystals, and assembly randomly oriented microspheroidal particles and equations for calculation of *α* using coordinates of particle tips.

Figure 4 shows schemes of top views of special cases of microspheroidal particle assemblies. On the left side a perfect colloidal nematic crystal is shown, in which long axes of all spheroidal particles are parallel. In the middle, there is the assembly, in which long axes of spheroidal particles are slightly misaligned with regard to the average orientation. For assemblies with such alignment, we propose calling them quasi-nematic colloidal crystals. The right scheme shows the top of the assembly of spheroids adsorbed flatly on the surface, but randomly oriented. The aforementioned cases could be recognized by measuring the angle (denoted *α*) between the long axis of each spheroid and Y axis of Cartesian coordinates. When *α* for every particle is equal to the average one, all particles are exactly oriented in the same direction (standard deviation; SD*_α_* = 0) and the arrangement is perfectly nematic. For a small standard deviation of *α* from its average value, the arrangement could be called quasi-nematic. It should be noted that *α* is equally probable in the range from 0° to 180° (for microspheroids with the same properties of both tips), SD*_α_* = 52°, and the average value of *α* is <*α*> = 90°.

**Figure 5:**
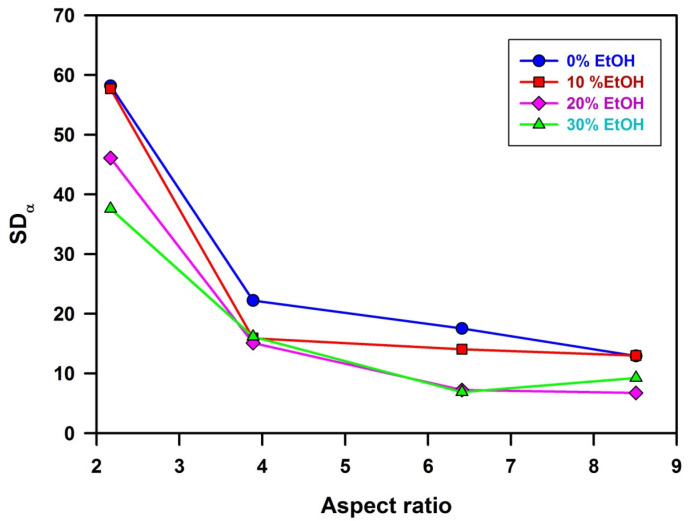
Plots of SD*_α_* as a function of *AR* for assemblies prepared from suspensions of P(S/PGL) microspheroids in water and water/ethanol media. Lines in the plots do not have any other meaning but are just to guide eyes.

Plots of SD*_α_* as functions of *AR* determined for particle assemblies formed from suspensions in water and water/ethanol mixtures are given in Figure 5. It is evident that for the smallest value of aspect ratio (*AR* = 2.17), standard deviations from the average angle *α* describing orientation of microspheroids in the surface layer of the assemblies are very large (from 38° to 58°, depending on ethanol content). Such large values of SD*_α_* characterize assemblies with a lack of ordered orientation of particles. However, for a slightly larger aspect ratio (*AR* = 3.89) SD*_α_* is much smaller (from 15° to 22°), indicating better orientation of microspheroids. For assemblies of microparticles that are more elongated, i.e., with *AR* 6.41 and 8.50, SD*_α_* is even smaller (in the range from 7° to 17°), indicating that these microspheroids form quasi-nematic assemblies. It is reasonable to assume that assemblies of microspheroids are formed not by simultaneous gathering but by sequential adsorption of microparticles, which align themselves to the particles at the surface of the growing assembly. The alignment is better for more elongated particles, enabling better interparticle contact. It should also be noted that better alignment of particles (lower values of SD*_α_*) when suspending medium with higher content of ethanol has been used since the effect is small and there might be various reasons for such behavior. It remains unexplained in this study. 

**Figure 6:**
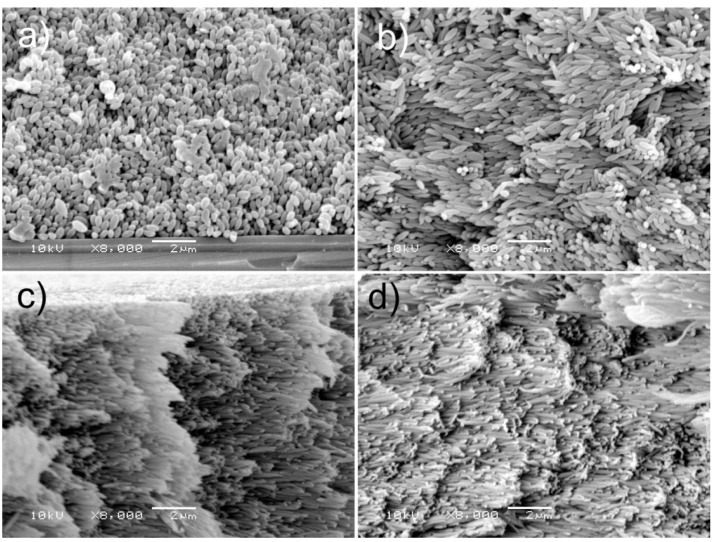
SEM microphotographs of fractures of the P(S/PGL) microspheroid assemblies prepared from particle suspensions in water/ethanol media containing 30% of ethanol. Particle aspect ratio: (**a**) 2.17, (**b**) 3.89, (**c**) 6.41, (**d**) 8.50.

The analysis presented above was related to the arrangement of microspheroids in the interfacial layer of particles assemblies. Thus, it was important to check whether microspheroids were also oriented in the interior of particle assemblies. Figure 6 shows SEM microphotographs of fractures of particle assemblies prepared using water/ethanol with 30% ethanol content as a suspending medium.

The picture in Figure 6a (microspheroids with *AR* = 2.17) is notable by a lack of preferential particle orientation. For microspheroids with *AR* = 3.89, the quality of angular orientation of microspheroidal particles is better. The best angular order was observed for assemblies of microspheroids with *AR* equal to 6.41 and 8.50. These observations conform to the results of the above-discussed studies of particle orientation on the surface of particle assemblies. It is worth noting that long axes of microspheres with a high degree of accuracy are parallel to the upper surface of particle assemblies. 

#### 3.2.3. Angle-Resolved Reflectance Spectroscopy Studies of Particle Assemblies

Since the investigated assemblies did not form the free-standing films but were prepared on solid supports, any studies based on the scattering of transmitted light posed significant problems. Thus, studies of reflectance were used in this work. Unfortunately, the equations, which could be used for light reflectance from nematic colloidal crystals, especially in which the particles are perfectly aligned in one direction, are not available. The multiple scattering-based models were developed for systems with scatterers randomly distributed in isotropic continuous phase, e.g., for emulsions or amorphous solids containing dispersed fillers. In colloidal assemblies prepared in this work, the situation is different. The continuous isotropic phase is absent. Perfect colloidal crystals made of spherical particles constitute another borderline case, for which the Bragg equation holds. The Bragg equation perfectly describes systems consisting of periodic alternating thin layers of polymers with different refractive indices and thickness characteristics for each type of polymer layer. SEM microphotographs of investigated particle assemblies revealed that at least particles with the highest *AR* were lying flat, parallel to the surface of the assembly, whereas their distribution along the long particle axis was irregular and the regularity of their distribution in perpendicular direction was fairly high. Therefore, we decided to check the plots of *λ_max_*^2^ versus *sin*^2^*θ* versus *sin*^2^*θ* and whether there is any monotonic change of parameter d and particle aspect ratio. We were very pleased to notice that experimentally determined points on plots *λ_max_*^2^ versus *sin*^2^*θ* could be fitted with a straight line and that their scatter is low (see Figure S7). We intend to check whether reflectance spectra could be deconvoluted to their components useful for determination of the distribution of the distances between the scattering planes in the future.

Angle-resolved reflectance spectra were recorded by changing the angle *θ* between beam of the incident light and normal to surface of the sample and measuring the intensity of reflected light as a function of its wavelength. For samples containing stacks of regularly distributed reflecting planes parallel to the sample surface, the following Bragg equation holds (Equation (7)):

(7) $$m^{2}\lambda_{max}^{2}=4d^{2}\left(n_{eff}^{2}-sin^{2}\theta \right)$$

where *m* denotes the order of diffraction, *λ_max_* the wavelength of maximum in reflectance spectrum, *d* the interplanar distance between diffracting surfaces, *n_eff_* the mean effective refractive index, and *θ* the angle between incident light and normal to the diffracting surface.

It should be noted that usually the spectra based on the first order diffraction are monitored. Thus, plots of $\lambda_{max}^{2}$ as a function of $sin^{2}\theta$ should be represented by the straight line with $slope=-4d^{2}$ and $intercept=4d^{2}n_{f}^{2}$. Using the aforementioned parameters, the interplanar distances and the mean effective refractive indices could be calculated.

Until today, the angle-resolved reflectance spectroscopy was used only for studies of colloidal crystals prepared from spherical particles [47,48,49,50,51,52]. The aforementioned colloidal crystals have a face-cubic centered (FCC) structure with the surface of the sample parallel to the plane defined by (111) Miller index. Thus, the interplanar distance in Equation (7) (*d*) denotes the distance between the adjacent (111) planes (*d*_111_).

**Figure 7:**
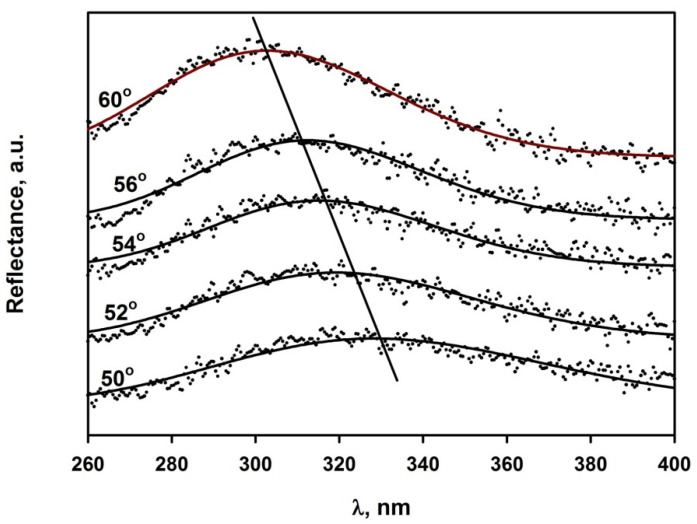
Reflectance spectra recorded at various angles of incidence light recorded for multilayer assemblies of P(S/PGL) microspheroids with *AR* = 3.89 deposited from suspensions in water/ethanol mixtures containing 20% of ethanol. Points were experimental. Lines were obtained by four parameter log-normal peak function fitting.

In the case of the assemblies of the P(S/PGL) microspheroids, which, as it is shown in Figure 3, are laterally oriented on surface of the samples, *d* has the meaning of the distance between the surface of the sample and the surface defined by the first inner layer of microspheroids. Typical plots of the reflectance spectra of the assembly of microspheroids are shown in Figure 7. Characteristic for these spectra was a large scatter of experimental points reflecting lower regularity of particles arrangement in quasi-nematic colloidal crystals of microspheroidal particles rather than almost-perfect colloidal crystals of microspheres. Values of $\lambda_{max}^{2}$ were determined from the four-parameter log-normal peak function fitted to experimental data. It should be noted that the surface of particle assemblies prepared from suspensions with 30% EtOH content had too many defects to obtain reflectance spectra with quality sufficient for enabling reliable determination of interplanar distances.

Plots of $\lambda_{max}^{2}$ as a function of $sin^{2}\theta$ prepared for assemblies of P(S/PGL) microspheroids are shown in Figure S7. Values of interplanar distances (*d*) were calculated from the slopes of straight lines of these plots using Equation (8).

(8) $$d=\frac{1}{2}\;\sqrt{-slope}$$

Their values are presented in Table 3. 

**Figure 8:**
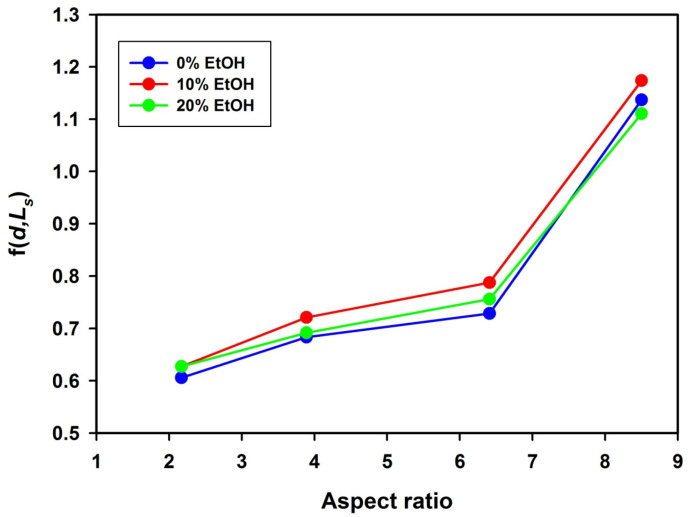
Plots of f(*d*,*L*_s_) as function of aspect ratio for microspheroidal P(S/PGL) particle assemblies prepared from particle suspensions in ethanol/water media.

Some information on packing particles in the assemblies can be obtained from analysis of the f(*d*,*L*_s_) parameter, defined as a ratio of interplanar distance *d* and particle short axis (*L*_s_). Plots of f(*d*,*L*_s_) as a function of aspect ratio are shown in Figure 8.

Figure 8 reveals that for each particular aspect ratio of investigated microspheroids, the values of f(*d*,*L*_s_) were very close, regardless of ethanol content in the suspending medium. This means that the f(*d*,*L*_s_) parameter was dependent mainly on aspect ratio and varied in a range from about 0.6 (for *AR* = 2.17) to 1.1 (for *AR* = 8.5). The increase of f(*d*,*L*_s_) with aspect ratio may look surprising. For the assembly of microspheroidal particles with *AR* = 4.3, M.J. Solomon [22] found f(*d*,*L*_s_) = 0.5 conforming to the body-centered tetragonal (BCT) lattice. Computer modeling based on Monte Carlo method revealed the formation of monoclinic spheroidal mC2 lattice with f(*d*,*L*_s_) = 0.6 [46]. 

**Figure 9:**
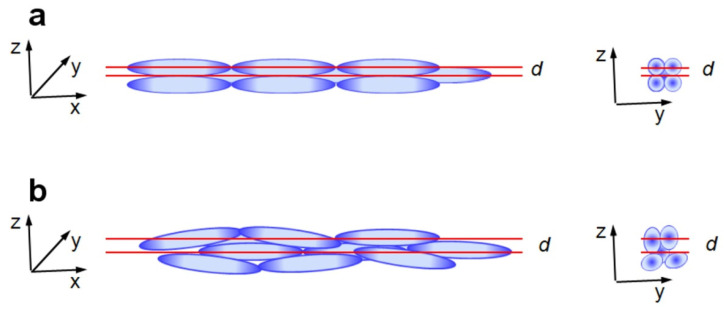
Schematic illustration of top layer of assembly of microspheroids with geometrical parameters of P(S/PGL)_s_3 (*AR* = 6.41, *L*_s_ = 221 nm): (**a**) body centered tetragonal colloidal crystal, for which interplanar distance *d* = 0.5*L*_s_; (**b**) quasi-nematic colloidal assembly of microspheroids with *d* = 0.756*L*_s_.

It has been also shown that for the smectic assembly, f(*d*,*L*_s_) must be equal 1 but SEM microphotographs recorded for assemblies investigated in this work exclude smectic arrangement [22,29]. However, the aforementioned observation (increase of f(*d*,*L*_s_) with increasing *AR* even to values of f(*d*,*L*_s_) exceeding 1) may be explained by taking into consideration that in investigated P(S/PGL) microspheroid assemblies, the long axes of particles are not perfectly parallel to the top surface plane of the sample and such misalignment would increase the interplanar distance. This effect may be seen in Figure 9, providing a comparison of interplanar distance for BCT colloid crystal of microspheroids and quasi-nematic colloidal assembly containing microspheroids tilted from the xy plane.

The consequence of larger interplanar distance is looser packing of microspheroids in z-direction, which might affect the mechanical properties of particle assemblies.

#### 3.2.4. Mechanical Properties of Particle Assemblies Determined by Nanoindentation

**Figure 10:**
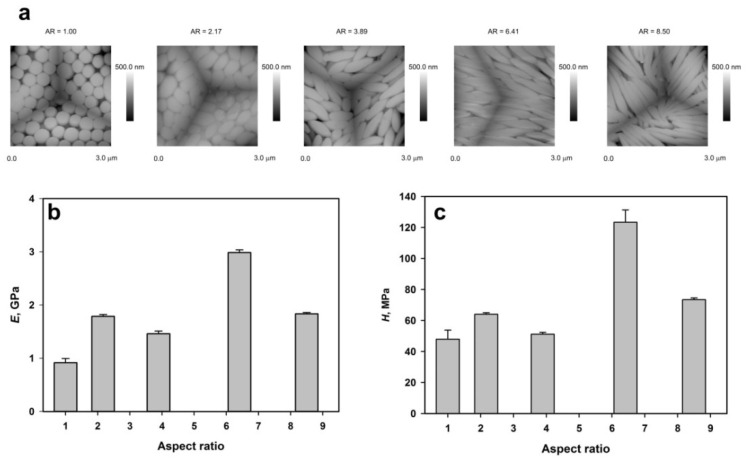
Mechanical properties of assemblies of microparticles determined using Berkovich nanoindenter; (**a**) imprint of nanoindenter in assembly of microparticles, (**b**) relation between modulus of elasticity and aspect ratio of microparticles, (**c**) relation between hardness and aspect ratio of microparticles. Plots were prepared using the number average and standard deviation values.

**Figure 11:**
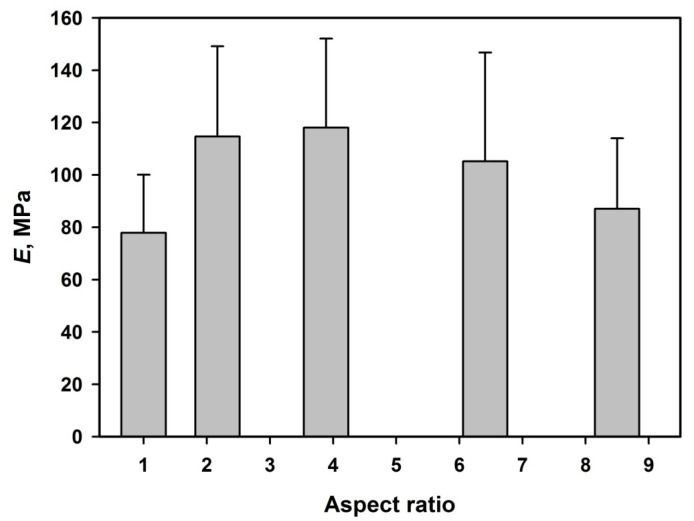
Modulus of elasticity determined using flat-punch nanoindenter as a function of aspect ratio of microparticles. Plots were prepared using the number average and standard deviation values.

Nanoindentation was used for studies of mechanical properties of various materials. There are even some reports on materials as soft as polymers [38]. Usually, polymer films were studied. However, in this paper, data describing mechanical properties of granular polymer assemblies built from spheroidal polymer particles are first presented (see Figure 10 and Figure 11). Figure 10a shows AFM microphotographs of an imprint of Berkovich nanoindenter in arrays of microspheroids with various aspect ratios. It should be noted that nanoindentation leads to angular reorientation, not of individual microspheroids but of whole domains of coherently oriented particles [38]. On the other hand, Monte Carlo simulation equivalent studies have shown that colloidal crystal assemblies with the SM2 (simple monoclinic with a basis of two ellipsoids) phase was formed by collective reorientation of particles in layers [53].

It was reasonable to expect that reorientation of domains consisting of microspheroids with larger aspect ratio would require larger forces and, indeed, there is a trend in relation to modulus of elasticity and aspect ratio (see Figure 10b).

Basic geometrical relations show that for microspheroids with the same volume, their surface growths with increasing aspect ratio making the shells of microspheroids, which was enriched in polyglycidol thinner. For polystyrene films, the measured value of hardness was 188 ± 27 MPa. Therefore, with increased aspect ratio and decreased thickness of microspheroid shells, it would be reasonable to expect hardness changing towards that of polystyrene. According to the plot in Figure 10c, such a trend has been observed, and for *AR* = 6.41, hardness reached 123 ± 8 MPa. As it has been already mentioned, the measurements with Berkovich nanoindenter provide information on local mechanical properties of studied material (roundness of the tip was r ≤ 20 nm) and the imprints show in Figure 10a that the arrangement of microspheroids was affected on the area about 10 μm^2^ large and involved a few tenths of particles.

On the other hand, in measurements using a flat-punch indenter, 100 μm diameter force was exerted on an area of about 7.9 × 10^4^ μm^2^ and affected thousands of microparticles.

Values of standard deviation and standard error of individual E and H of all spheroid samples were calculated according to the equations (Equations (S4)–(S6)) presented in Analytical Methods S2. The statistical analysis using *t* Student test of obtained results from nanoindentation studies is described in Analytical Methods S2.

The aforementioned analyses performed for measuring local (Berkovich) elastic moduli and hardness revealed that the differences between assemblies of nanoparticles with *AR* = 1, 2.17, 3.89, 6.41, and 8.50 were statistically meaningful. In the case of measurements with the flat-punch indenter (contact with several hundreds of microspheroids), similar differences were statistically insignificant only for five pairs. Thus, conclusions concerning trends of modulus of elasticity determined using flat-punch nanoindenters are problematic. 

It should be noted that the modulus of elasticity measured using the flat-punch nanoindenter is more than an order of magnitude smaller than the one measured using the Berkovich nanoindenter. Apparently, the measurements with Berkovich nanoindenter with a roundness of nanoindenter provide information mostly on single microspheroids, whereas the measurements with the flat-punch nanoindenter provide information on properties of assemblies of not very tightly packed microspheroids, which may undergo not only compression but also bending perpendicular with respect to the particles long action. It is worth noting that increasing interplanar distance in particles assemblies with increasing particles aspect ratio (see Section 3.2.3), indicating less dense packing of more elongated microspheroids, conforming to observation of the decreasing modulus of elasticity with the increasing aspect ratio.

## 4. Conclusions

Uniaxial elongation of polymer microspheres proceeding with maintenance of their volumes produces microspheroids, which, in addition to three positional degrees of freedom, characterizing microspheres have new degrees of freedom related to angular orientation of particle long axis. The aforementioned situation affects the order in particle assemblies, particularly when particles have interfacial layers enriched in polyglycidol, making them inter-particle “sticky” due to intermolecular polyglycidol interactions, presumably via hydrogen bonding [36,47]. It is not surprising that geometrical restrictions (jamming) impose that the neighbor spheroids assume orientation in dense assemblies, with particles’ long axes being close to parallel, and that their assemblies can be called quasi-nematic colloidal crystals. However, studies described in this work revealed that such behavior was observed for assemblies of microspheroids, with aspect ratio significantly exceeding 2.17. For assemblies of microspheroids with *AR* = 2.17, their orientation was completely chaotic—for 3.89 ≤AR ≤8.50 standard deviation of the microspheroid long axis from its average value decreased, and *AR* = 8.50 was in the range from 6.7° to 13° only, depending on ethanol content in the suspending medium, from which the assemblies were prepared. Angular ordering of microspheroids was observed not only on the surface of particle assemblies and in the first two layers of microspheroids but across the whole assembly samples from 40 to 70 μm thick. Nanoindentation studies revealed that the local modulus of elasticity (forces affecting only few microspheroids during nanoindentation with sharp Berkovich tip) was about an order of magnitude larger than modulus of elasticity measured for deformation of circular area of 100 μm diameter of the microspheroidal assemblies.

## Figures and Tables

**Table 1:** Basic characteristics of spheroidal particles and elongation parameters.

Symbol of Particles	Final Length of PVA Film Stripe, mm	PVA Film Elongation, %	*AR* of Spheroids	Length of Long Axis, nm	Std. Dev.	Length of Short Axis, Nm	Std. Dev.
P(S/PGL)_s_1	90	150	2.17	606	0.066	279	0.025
P(S/PGL)_s_2	120	200	3.89	935	0.116	240	0.026
P(S/PGL)_s_3	170	283	6.41	1419	0.148	221	0.025
P(S/PGL)_s_4	220	366	8.50	1620	0.153	190	0.023

**Table 2:** Chemical composition of the P(S/PGL) spherical and spheroidal particles and surface fraction of adsorbed PVA.

Symbol of Particles	*AR*	C%, from XPS	O%, from XPS	f_PGL_, M, Calculated from XPS Data	PVA Irreversibly Adsorbed on Microspheres and Microspheroids *, % (wt/wt)
P(S/PGL)_m_	1.00	92.28	7.43	0.216	-
P(S/PGL)_m_-PVA	1.00	86.89	12.89	-	5.0
P(S/PGL)_s_3	6.41	91.71	8.14	-	5.8

* fraction of poly(vinyl alcohol) absorbed on the surface of spherical or spheroidal particles after the removal of unbound fraction by repeated centrifugation, determined using 5-DTAF labeled PVA.

**Table 3:** Characteristics of spheroidal assemblies determined by angle-resolved UV spectroscopy.

Symbol of Particles	Short Axis of Microspheroid, nm	EtOH Content in Suspending Water/Etanol Medium, % v/v	Interplanar Distance (*d*), nm
P(S/PGL)_s_1	279	0	169
		10	175
		20	175
P(S/PGL)_s_2	240	0	164
		10	173
		20	166
P(S/PGL)_s_3	221	0	164
		10	174
		20	167
P(S/PGL)_s_4	190	0	216
		10	223
		20	211

## Data Availability

The data presented in this study are available on request from the corresponding author.

## Supplementary Materials

The following supporting information can be downloaded at: https://www.mdpi.com/article/10.3390/polym14224859/s1, Methods S1: Procedure for preparation of poly(styrene/α-tert-butoxy-ω-vinylbenzylpolyglycidol) (PS/PGL) spheroidal microparticles, Preparation of silicon and glass plates for studies of particles deposition; Table S1: Basic properties of microspheres (P(S/PGL)_m_); Analytical Methods S2; Table S2. Values of t parameter for differences of average values of elastic modulus of assemblies of microspheroids with different aspect ratio (*AR*) measured using Berkovich and flat-punch indenters and hardness using the Berkovich indenter; Figure S1: ^1^H NMR spectrum of PGL with protected hydroxyl groups (solvent: CDCl_3_); Figure S2: ^1^H NMR spectrum of PGL macromonomer with deprotected hydroxyl groups (solvent: D_2_O); Figure S3: GPC chromatogram of PGL with protected hydroxyl groups (eluent: methylene chloride); Figure S4: Dependence of the stretching ratio of microparticles on stretching ratio of the PVA film; Figure S5: C1s spectra after signal deconvolution of (a) pristine P(S/PGL)_m_ particles; (b) P(S/PGL)_m_ particles after removal from PVA film; (c) P(S/PGL)_s_ spheroids; Figure S6: Representative dependence of zeta potential of P(S/PGL) spherical and spheroidal particles on concentration of NaCl in aqueous particles suspensions; Figure S7: Dependencies of *λ_max_*^2^ versus *sin*^2^*θ* determined for spheroidal assemblies deposited from aqueous suspensions containing ethanol in the range 0–20 vol%; Table S3: Water contact angle measurements of particles assemblies deposited from aqueous suspensions with various concentration of ethanol [54,55,56,57].
