# Supplementary material for: New Class of Polymer Materials—Quasi-Nematic Colloidal Particle Self-Assemblies: The Case of Assemblies of Prolate Spheroidal Poly(Styrene/Polyglycidol) Particles
Source: Polymers (Basel). 2022 Nov 11;14(22):4859. doi: 10.3390/polym14224859 (PMC9698966; doi:10.3390/polym14224859)
Supplement: Supplementary file 1 [file polymers-14-04859-s001.zip › polymers-1956098-supplementary.pdf]

New class of polymer materials - quasi-nematic colloidal particle self-assemblies. The case of assemblies of prolate spheroidal poly(styrene/polyglycidol) particles

Damian Mickiewicz[a], Mariusz Gadzinowski[a], Tomasz Makowski[a], Witold Szymański[b], Stanislaw Slomkowski[a], Teresa Basinska[a]**

[a] Division of Functional Polymers and Polymer Materials, Centre of Molecular and Macromolecular Studies, Polish Academy of Sciences, H. Sienkiewicza 112, 90-363 Lodz, Poland;

[b] Institute of Materials Science and Engineering, Technical University of Lodz, B. Stefanowskiego 1/15, 90-924 Lodz, Poland;

SUPPLEMENTARY MATERIALS

S1. Methods.

Procedure for preparation of poly(styrene/α-tert-butoxy-ω-vinylbenzylpolyglycidol) (PS/PGL) spheroidal microparticles.

Preparation of spheroidal particles (PS/PGL) consisted of the following steps:

i) synthesis of α-tert-butoxy-ω-vinylbenzyl-polyglycidol (PGL) macromonomer,

ii) synthesis of $P(S/PGL)_m$ microspheres using styrene and PGL macromonomer,

iii) preparation of spheroidal particles $P(S/PGL)_s$ from the spherical ones. Poly(styrene/α-*tert*-butoxy-ω-vinylbenzyl-polyglycidol) ($P(S/PGL)_s$) spheroidal microparticles were produced by stretching poly(vinyl alcohol) (PVA) films containing embedded $P(S/PGL)_m$ microspheres.

Synthesis of α-tert-butoxy-ω-vinylbenzyl-polyglycidol macromonomer.

Briefly, the process consisted of three steps: synthesis of 1,1-ethylethoxyglycidyl ether in reaction of glycidol and ethyl vinyl ether[1], anionic polymerization of 1,1-ethylethoxyglycidyl ether initiated with potassium tert-butoxide and terminated with p-chloromethylstyrene. Hydrolysis of ethyl ethoxy groups in poly(1 ethylethoxy-glycidyl ether) was performed using $AlCl_3$ x 6 H_2O, according to procedure described by Namboodiri[2] and subsequently applied by Halacheva et al.[3] for the removal of ethyl ethoxy groups from

polyglycidol macromolecules. The process yielded poly(α-*tert*-butoxy-ω-vinylbenzyl-polyglycidol) macromonomer. The macromonomer structure, molecular weight and dispersity were confirmed by ^{1}H NMR spectra of macromonomer before and after hydrolysis of ethyl ethoxy groups and GPC traces, respectively. The corresponding ^{1}H NMR spectra and GPC chromatogram of PGL with protected hydroxyl groups are presented in Figure S3.1, Figure S3.2 and Figure S3.3, respectively.

In the ^{1}H NMR spectrum of poly[*α*-*tert*-butoxy-*ω*-vinylbenzyl-poly(1-ethylethoxyglycidyl ether)], the following signals were observed: 7.38 (m) protons in phenyl ring; 6.71 (m) proton in vinyl group; 5.25 (d) and 5.80 (d) $–CH_2$; 4.60 (d) $–C_6H_5–CH_2–O$; 3.40-3.75 (m) –OCH, $–OCH_2$; 1.17 (m) $–C(CH_3)_3$, $–CHCH_3$, $–OCH_2CH_3$, whereas after hydrolysis of ethyl ethoxy groups in ^{1}H NMR spectrum signals with the following chemical shifts (in ppm), assigned to protons in PGL macromolecule have been registered: 7.38 (m) protons in phenyl ring; 6.71 (m) proton in vinyl bond; 5.78 (d) and 5.23 (d) $=CH_2$; 4.60 (d) $–C_6H_5–CH_2–O$; 3.45-3.64 (m)–CH<, $–CH_2O–$; 1.12 (s) $−C(CH_3)_3$. The spectrum did not contain signals of ethyl ethoxy groups, which confirmed the full removal of the blocking components. Integration of $-C(CH_3)_3$ (at 1.12 (s)) and of –CH< and $–CH_2O−$ (overlapping signals in the range 3.45-3.64) allowed the calculation of the M_n of the PGL macromonomer.

The number average molecular weight and molecular weight dispersity of the two macromonomer samples, determined by GPC (with calibration for polystyrene) before deprotecting hydroxyl groups by hydrolysis of ethoxy ethyl groups, were equal 8030 (DI=1.36), respectively (Figure S3.1). It was already shown, that deprotection performed according to the abovementioned protocol, occurs without chain scission.[3] Thus, it is justified to assume that the M_n of the synthesized *α*-*tert*-butoxy-*ω*-vinylbenzyl-polyglycidol macromonomer before and after hydrolysis of ethyl ethoxy groups were equal to 6300 and 3370, respectively. These values were found by end-group ($(CH_3)_3C–$) and main-chain groups ($–CH_2CH(CH_2OH)O–$) analysis of the macromonomer ^{1}H NMR spectra (see Figure S3.1 and Figure S3.2). It is worth to know, that deprotection changes molar mass of PGL macromonomer but not the distribution of the degree of polymerization and thus also the distribution of molar masses.

Procedure for preparation of poly(styrene/α-tert-butoxy-ω-vinylbenzylpolyglycidol) ($P(S/PGL)_m$) microspheres.

Poly(styrene/*α-tert*-butoxy-ω-vinylbenzylpolyglycidol) $(P(S/PGL))_m$ microspheres were synthesized by the emulsion polymerization of styrene and α-*tert*-butoxy-ω-vinylbenzylpolyglycidol macromonomer (PGL). The details of the synthesis were described in our earlier reports.[4,5] Briefly, the PGL macromonomer was dissolved in 120 ml of distilled water, and then styrene (10 g) was placed in a glass reactor, degassed by purging with argon and stirred at room temperature. After 60 minutes of stirring, the temperature was raised to 70 °C and 0.216 g of $K_2S_2O_8$ was added. The polymerization was carried out under argon at 70 °C for 27 h with stirring at 400 rpm. For preparation of $P(S/PGL)_m$ 0.250 g of PGL macromonomer was added to the polymerization mixture.

Then, the microspheres were purified by centrifugation, washed with 10^{-3} M HCl and then washed with new portions of water. The washing steps were repeated four times. The diameters of the microspheres were determined by analysis of the scanning electron microscopy (SEM) microphotographs. The number average diameters of microspheres (D_n) and diameter dispersity parameter (D_w/D_n) were calculated on the basis of measurements of the diameters of ca. 500 microspheres (randomly chosen from different microphotographs). The characteristic features of $P(S/PGL)_m$ particles used for manufacturing of spheroids illustrates Table S1.

Table S1. Basic properties of microspheres ($P(S/PGL)_m$).

Symbol of microspheres	Styrene:PGL, mol:mol Monomers feed	M_n of PGL, M_w/M_n	DP of PGL (^{1}HNMR)	D_n, nm, D_w/D_n (SEM)	D_h, nm PDI (PCS)	$f_{PGL\text{-}total}$, mol,(EA)[1]	$f_{PGL\text{-}surface}$, mol (XPS)
$P(S/PGL)_m$	$1:7.62x10^{-4}$	3370; 1.36	42	408; 1.04	430	0.0003	0.216

[1]fraction of polyglycidol in the whole microspheres calculated from elemental analysis (EA).

Procedure for preparation of poly(styrene/α-tert-butoxy-ω-vinylbenzylpolyglycidol) (P(S/PGL)) spheroidal particles.

The P(S/PGL) microspheres (4.485 g of 4 wt% suspension) were mixed with aqueous solution of poly(vinyl alcohol (12.5 % w/w). Then, 22.98 g of the mixture was transferred to a PTFE mold with the size 7x9 cm and stored at 22 °C for 4 days to evaporate water. The dried films were removed from the mold and cut into strips of size 1x8 cm. Then, the thickness of the strips every 1 cm along their length was measured using a thickness tester with an accuracy of ±1 μm (Sylvac μs 229). In the next step, the PVA strips with embedded particles were uniaxially stretched in a chamber with controlled temperature using an Instron apparatus (ITW). The size of the strips that underwent elongation were 1x6 cm plus an additional 1 cm on each side for catching by

clamps. Then, the strips were elongated with a constant speed of 10 mm/min until the required length of the strip was obtained. When the temperature in the heating chamber decreased to approximately 50 °C, the stretched strips of the PVA of film were removed from the clamps and cooled to room temperature. Next, the length and thickness of the film strips were measured again. In the final step, the particles were recovered by dissolving the PVA matrices in deionized water followed by repeated centrifugation in a centrifuge (380R, MPW, Poland) and multiple exchange of supernatant for deionized water. Every 2-3 washings, the supernatant was checked for the presence of PVA by measuring its surface tension. Eventually, the washing procedure was repeated, when the difference between surface tension of the deionized water and supernatant decreased below 0.5 %, indicating that the supernatant was PVA-free.

Preparation of silicon and glass plates for studies of particles deposition.

Glass supports were cut into smaller pieces, usually ca. 1.5 x 1.5 cm, and subsequently washed by incubation in isopropanol/KOH mixture for 2 h, then abundant washing with di. water and drying under stream of nitrogen.

Silicon wafers were cut into smaller pieces with the size 3 x 3 or 1 x 1 cm each and thoroughly washed using methylene chloride and then, isopropanol. Finally, Si wafers were washed with di. water, dried under stream of nitrogen. In the following step, the drops of particles suspensions (microspheres or microspheroids) were deposited on the silicon wafers and after subsequent evaporation of solvent (water or water/ethanol mixtures) the particles arrangements were analyzed using SEM, AFM, goniometer or nanoindenter.

S2. Analytical Methods.

^{1}H NMR spectra of PGL macromonomer samples dissolved in $CDCl_3$ were recorded on a Bruker, 200 MHz (Karlsruhe, Germany).

Gel permeation chromatography (GPC) traces were recorded using a system composed of a 1100 Agilent isocratic pump (Santa Clara, CA USA), a photometer MALLS DAWN EOS (Wyatt Technology Corporation, Santa Barbara, CA), and a differential refractometer K-2300 (Knauer). ASTRA 4.90.07 software (Wyatt Technology Corporation) was used for data collecting and processing. Two TSK Gel columns (G 2000 H and

G 6400 H) were used for separation. Samples were injected as solutions in methylene chloride. The volume of the injection loop was 100 μL. Methylene chloride was used as the mobile phase at a flow rate of 0.8 ml/min.

Scanning electron (SEM) microphotographs were recorded using a JEOL 5500LV apparatus (Akishima, Japan). The exemplary SEM images of each particles sample were transformed into two-dimensional FFT images using Fourier Painter v. 1.0.1.0 software.

AFM images were recorded under ambient atmosphere using Nanosurf Flex Axiom with controller C3000, (Nanosurf AG, Switzerland). The analysis was performed using tapping mode (TM). The probes were commercially available rectangular silicon cantilevers (RTESP Vecco) with a nominal radius of curvature of less than 10 nm (symmetric geometry), a spring constant of 40 N/m, and a resonance frequency of 300 kHz. The images were recorded with a resolution of 512 x 512 data points. The image analysis was performed using SPIP Image Metrology software (Denmark).

Surface tension measurements of supernatants after individual washings of particles were performed using a TSD01 tensiometer (Gibertini Elettronica, Milano, Italy). For the determination of surface tension, the method with the du Noüy ring was applied.

X-ray photoelectron spectroscopy (XPS) experiments were performed in a PHl 5000 VersaProbe - Scanning ESCA Microprobe (ULVAC-PHI, Japan/USA) instrument at a base pressure below 5×10^{-9} mbar. Monochromatic AlKa radiation was used, and the X-ray beam, focused to a diameter of 100 micrometer, was scanned on a 250×250 micrometer surface at an operating power of 25 W (15 kV). Photoelectron survey spectra were acquired using a hemispherical analyzer with a pass energy of 117.4 eV and a 0.4 eV energy step. Core-level spectra were acquired at a pass energy of 23.5 eV with a 0.1 eV energy step. All spectra were acquired with 90° between the X-ray source and the analyzer. Low-energy electrons and low-energy argon ions were used for charge neutralization. After subtraction of the Shirley-type background, the core-level spectra were decomposed into their components with mixed Gaussian-Lorentzian (30:70) shape lines using the CasaXPS software and sensitivity factors supplied by PHI.

Determination of zeta potentials of spherical and spheroidal P(S/PGL) microparticles.

Electrophoretic mobilities (μ) of P(S/PGL) microspheres were measured using Nano Sizer ZS (Malvern) in a cell equipped with electrodes, to which a controlled potential (up to 400 V) was applied. The electrophoretic mobility of an individual particle depends on surface charge (q), field strength (E), viscosity of the medium (η), and is expressed by **Equation (S1)**:

$$u = \frac{q \cdot E}{6\pi \cdot \eta \cdot r_h} \tag{S1}$$

where r_h denotes hydrodynamic particle radius. Substitution of q in **Equation (S2)**:

$$q = \zeta \cdot 4\pi \cdot \varepsilon \cdot r \tag{S2}$$

gives relation between electrophoretic mobility and electrokinetic potential (ζ) (**Equation (S3)**):

$$u = \frac{2}{3} \cdot \frac{\varepsilon \cdot \zeta \cdot E}{\eta} \tag{S3}$$

The electrophoretic mobility measurements were performed for 3 ml samples of aqueous suspensions containing NaCl in a range 0.0-1x10^{-1} M at 25 ^{o}C. Temperature dependence of viscosity and dielectric constant was taken into account during data treatment. The average of 10 measurements recorded at required number of counts (usually ca. 1500 kcounts/s) was taken for analysis of zeta potential.

Water contact angle measurements deposited on spherical or spheroidal particles assemblies

Water contact angle measurements were performed using Phoenix-300 goniometer (SEO Surface Electro Optics, Korea). The measurements were performed using 2 μl of di. water deposited on freshly prepared particles assemblies. The obtained contact angle value was taken from averaged three independent contact angle measurements taken immediately after deposition drop of water.

Statistical analysis of nanoindentation results

For indentation studies, 3 assembly samples were prepared for each type (aspect ratio) of nanoparticles. For each of them 6-7 indentation processes were monitored and analyzed. Altogether data for N = 20 indentations on randomly chosen places of the samples were recorded for assemblies of each type of microparticles (E_i, H_i, I = 1, … ,20). The number average values for assemblies of particles with a given aspect ratio (AR) were calculated as follows (**Equation (S4)**):

$$X(AR) = \frac{\sum_{i=1}^{20} X_{\mathrm{i}}(AR)}{N = 20}$$
(S4)

Where for symbol X should be replaced by E or H, correspondingly.

Values of standard deviation [s_X(AR)] and standard error [SE_X(AR)] were calculated using formulae (**Equation (S5)** and **(S6)**):

$$s_X(AR) = \sqrt{\frac{\sum_{i=1}^{N}[X_i(AR) - X(AR)]^2}{(N-1) = 20}}$$
(S5)

$$SE_X(AR) = \frac{s_X(AR)}{\sqrt{N}}$$
(S6)

Plots were prepared using the number average and standard deviation values.

Checking whether the differences between pairs of elastic modulus (or hardness) determined for assemblies of particles with aspect ratios AR1 and AR2 were meaningful was performed using the *t* Student test. Values of parameter *t* were calculated from formula (**Equation (S7)**):

$$t = \frac{|X(AR1) - X(AR2)|}{\sqrt{\frac{{s_{X(AR1)}}^2}{N_1} + \frac{{s_{X(AR2)}}^2}{N_2}}}$$
(S7)

For $t_{\mathrm{critical}} < t_{\mathrm{calculated}}$ it was assumed that the tested difference is statistically meaningful. For t_{critical} was used value (2.025) corresponding to degree of freedom $N_1+N_2-2 = 38$ and level of confidence 0.025.

Values of t for the above-mentioned conclusions are given in the **Table S2** shown below.

Table S2. Values of t parameter for differences of average values of elastic modulus of assemblies of microspheroids with different aspect ratio (AR) measured using Berkovich and flat-punch indenters and hardness using the Berkovich indenter.

AR of sample 1	AR of sample 2	t values for determination of significance of differences between averages of elastic modulus of spheroidal particle assemblies measured using Berkovich indenter	t values for determination of significance of differences between averages of hardness of spheroidal particle assemblies measured using Berkovich indenter	t values for determination of significance of differences between averages of elastic modulus of spheroidal particle assemblies measured using flat-punch indenter
1.00	2.17	44.9142	12.0398	4.0147
1.00	6.41	25.6901	2.3947	4.4278
1.00	8.50	97.0218	34.0982	2.5900
1.00	3.89	49.0351	18.9894	1.1745
2.17	3.89	23.9314	36.7199	0.3141
2.17	6.41	86.1619	33.1966	0.7866
2.17	8.50	4.8746	28.7992	2.8242
3.89	6.41	93.6358	40.1999	1.0740
3.89	8.50	29.4479	59.6801	3.1985
6.41	8.50	89.2141	27.8534	1.6361

S3. Supplementary figures and table.

^{1}H NMR spectra of PGL macromonomer before and after deprotection of hydroxyl groups.

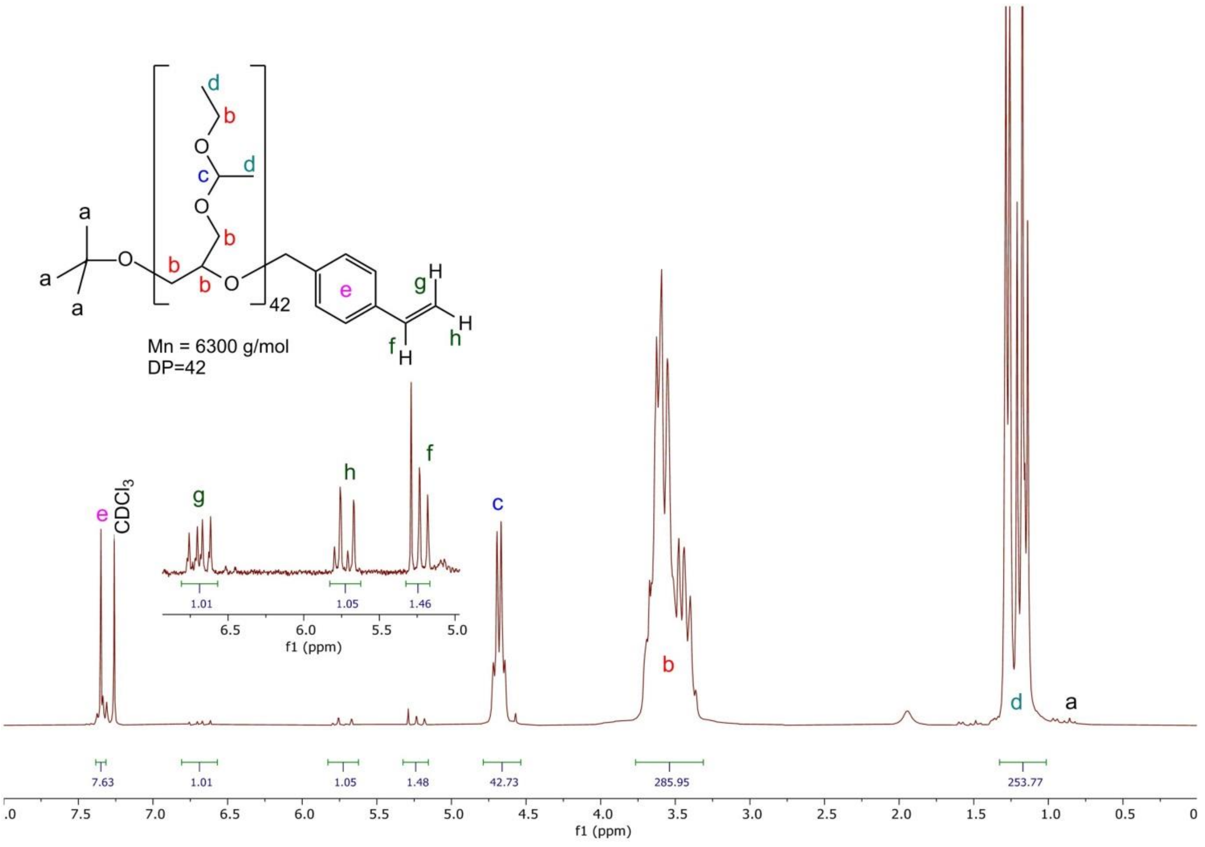

Figure S1. ^{1}H NMR spectrum of PGL with protected hydroxyl groups (solvent: $CDCl_3$).

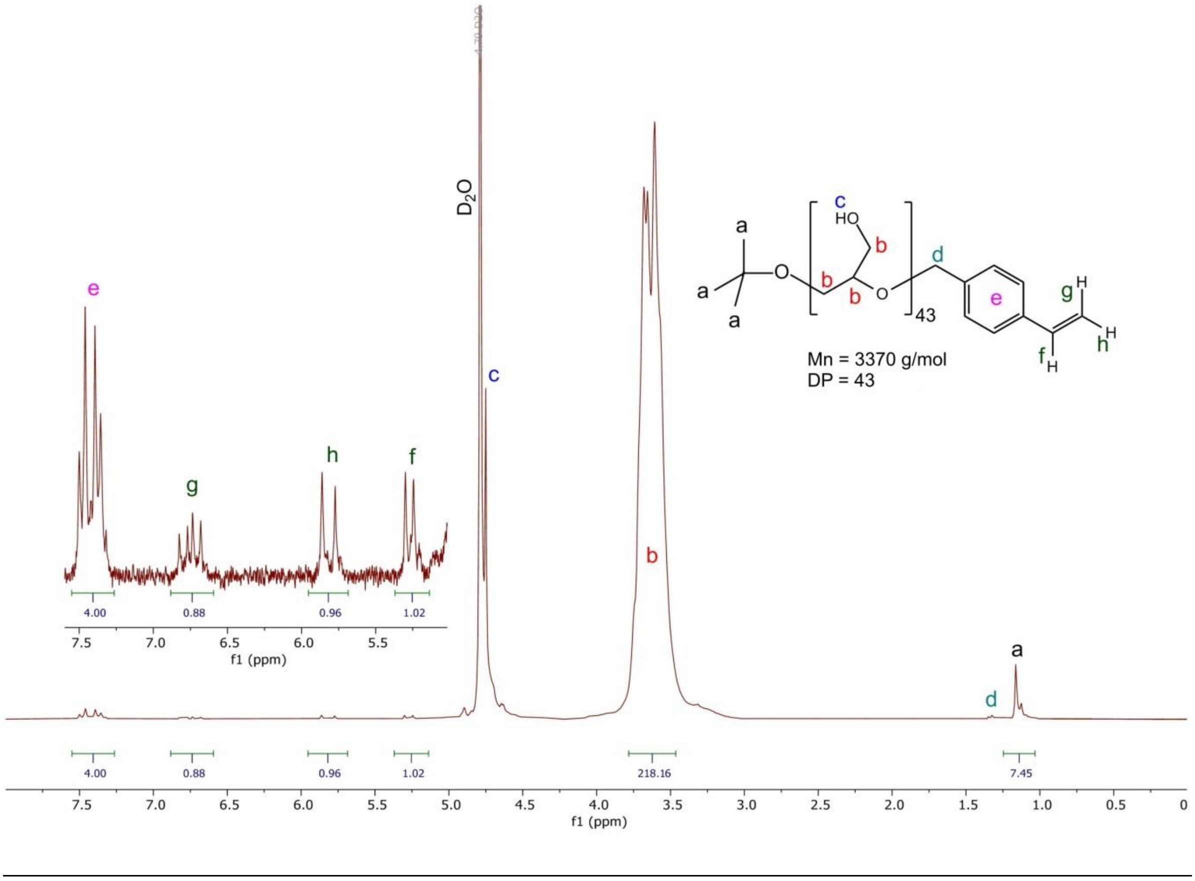

Figure S2. ^{1}H NMR spectrum of PGL macromonomer with deprotected hydroxyl groups (solvent D_2O).

Chromatogram (GPC) of PGL macromonomer with protected hydroxyl groups.

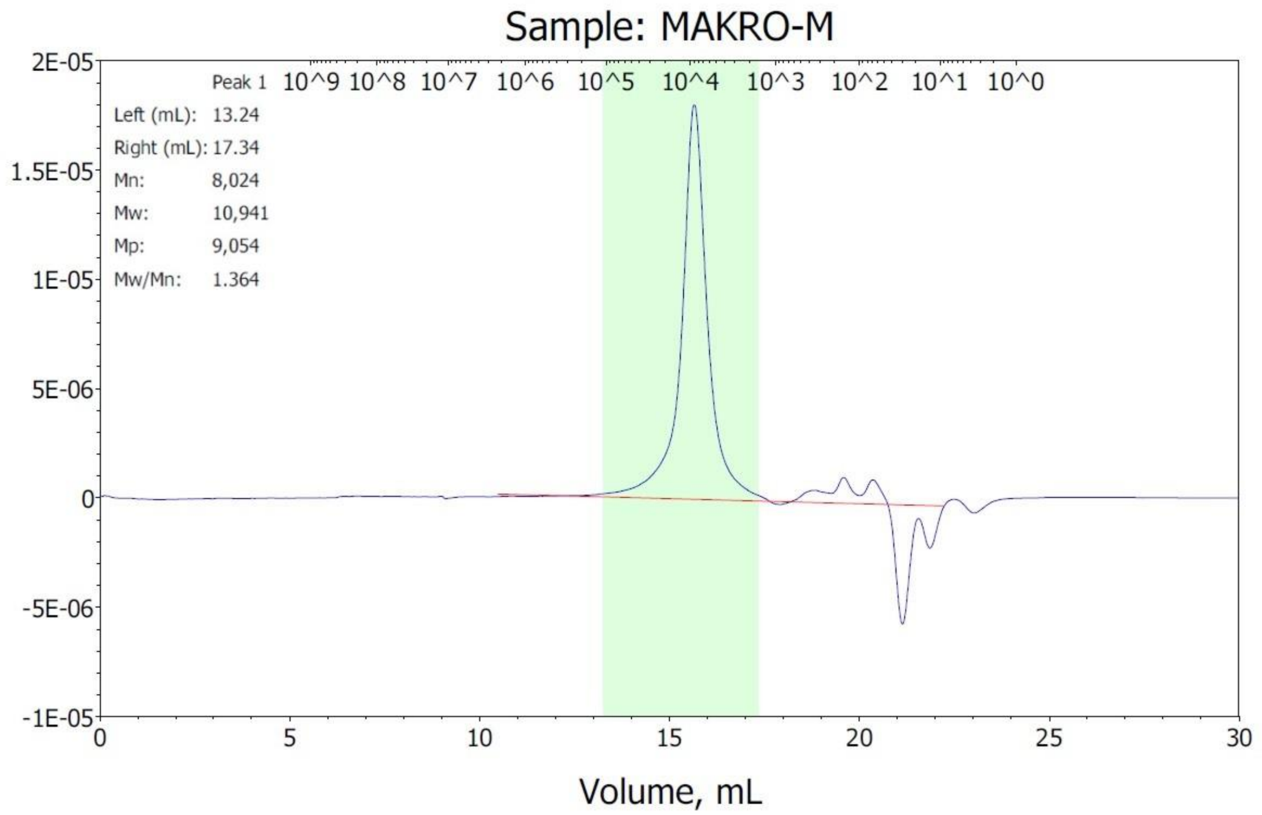

Figure S3. GPC chromatogram of PGL with protected hydroxyl groups (eluent: methylene chloride).

Relation between stretching ratio of the microspheres and elongation of the film.

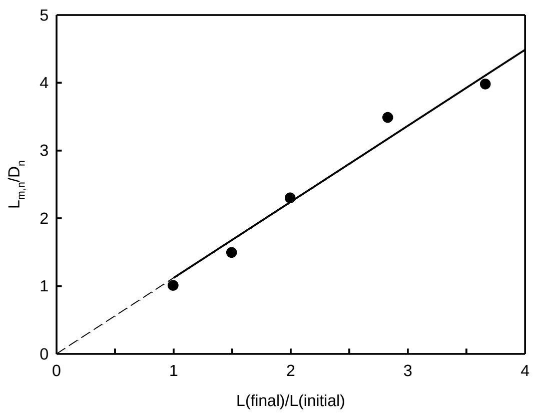

Figure S4. Dependence of the stretching ratio of microparticles on stretching ratio of the PVA film. $L_{m,n}$ denotes longer axis of spheroids, D_n - number average diameter of P(S/PGL) microspheres determined from SEM. Stretching ratio of PVA film is expressed by ratio of final to initial stripe of film length (equal 60 mm).

C1s XPS spectra after deconvolution of signals deriving from carbon atoms in various chemical environments.

a.

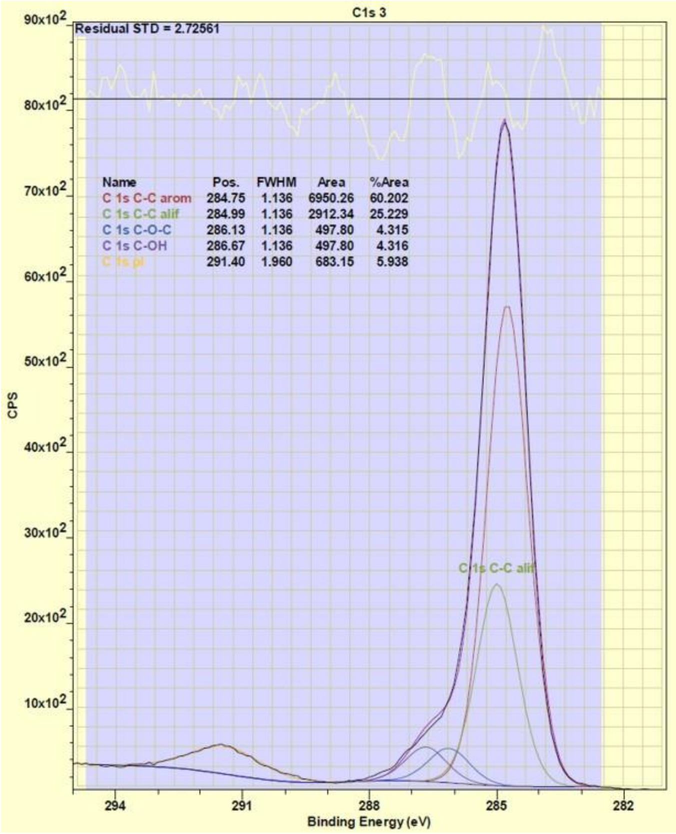

b.

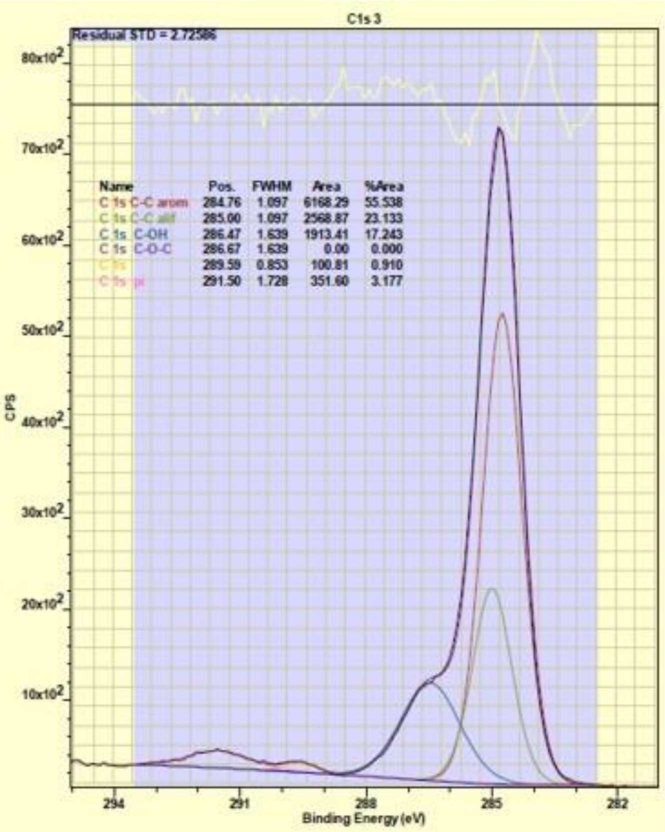

c.

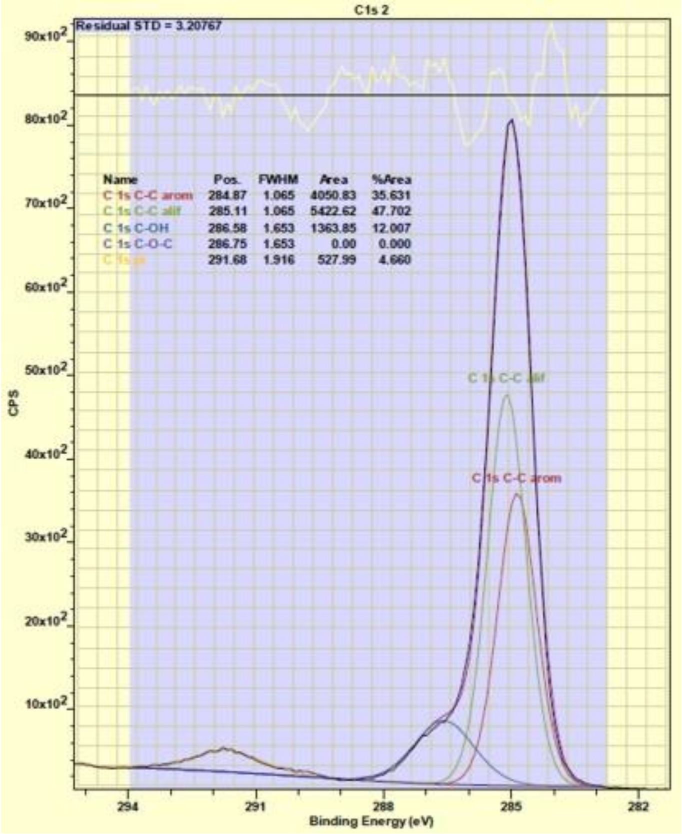

Figure S5. C1s spectra after signal deconvolution of a) pristine $P(S/PGL)_m$ particles; b) $P(S/PGL)_m$ particles after removal from PVA film; c) $P(S/PGL)_s3$ spheroids.

Dependence of zeta potential on concentration of NaCl in suspensions of spherical and spheroidal particles with various aspect ratios.

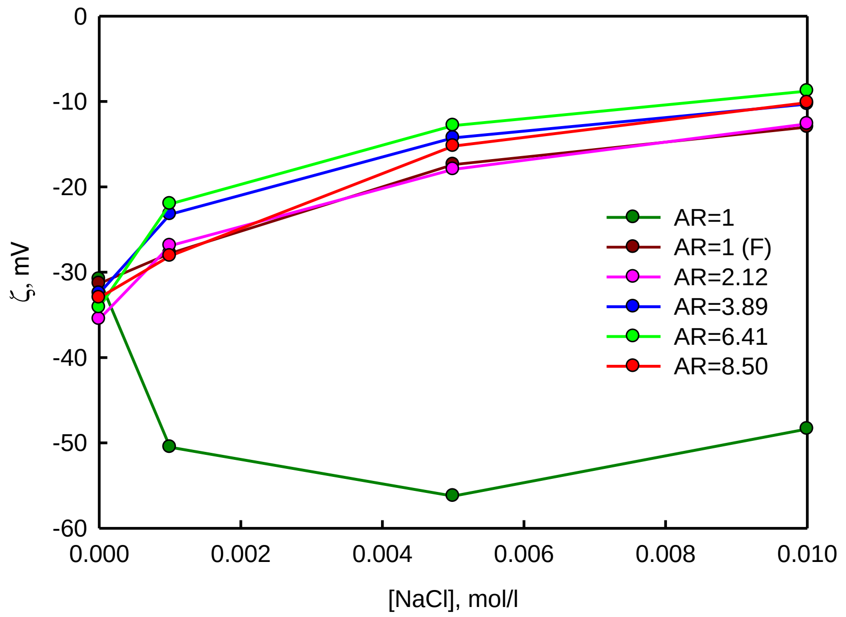

Figure S6. Representative dependence of zeta potential of P(S/PGL) spherical and spheroidal particles on concentration of NaCl in aqueous particles suspensions.(AR=1 denotes pristine spherical particles, whereas AR=1 (F) denotes spherical particles embedded in PVA film, incubated in 120 ^{o}C for 30 min, released from film and finally washed out from the PVA.)

UV-Vis angle-resolved reflectance studies of colloidal arrays.

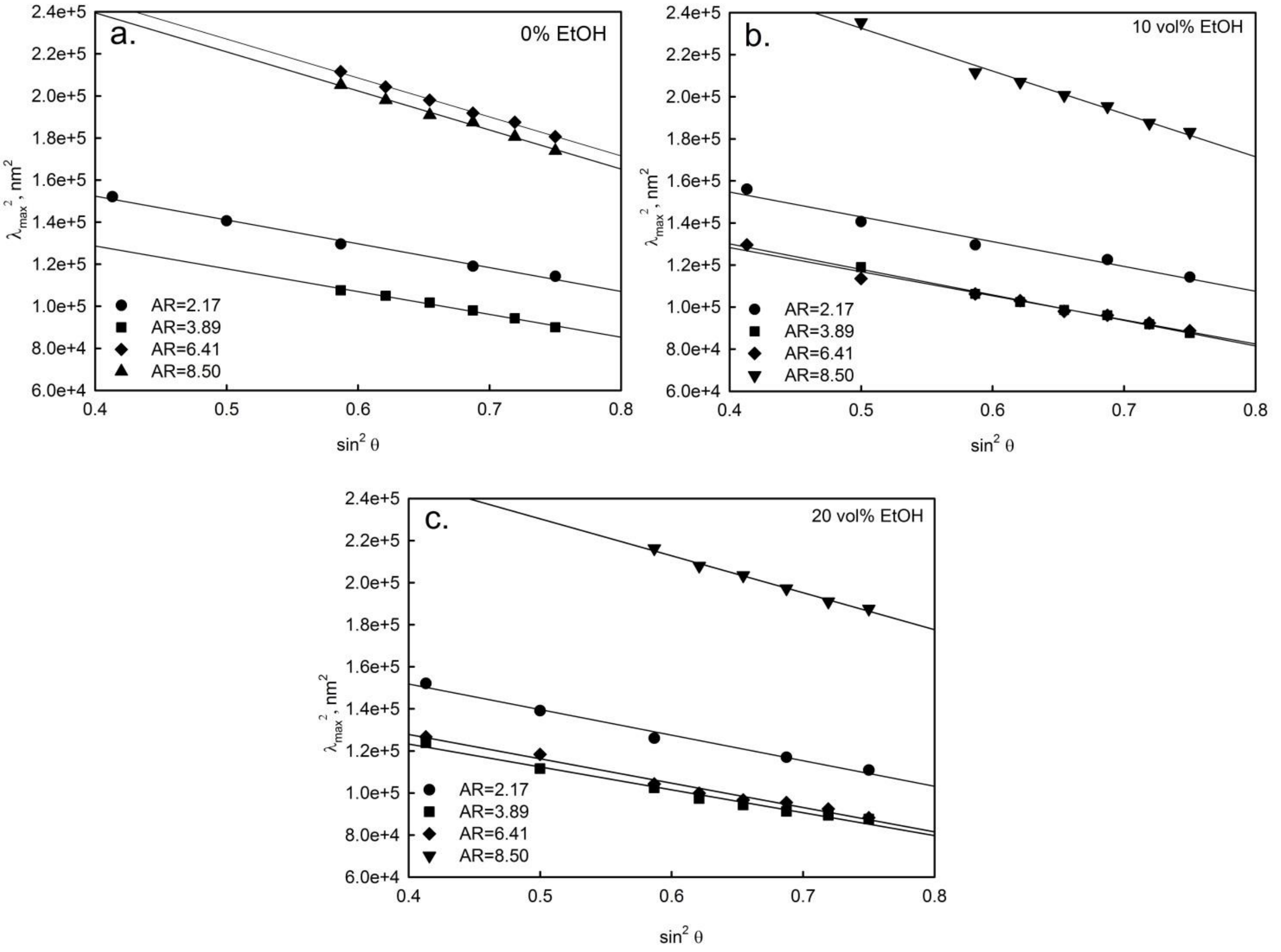

Figure S7. Dependencies of λ_{max}^2 versus $\sin^2\theta$ determined for spheroidal assemblies deposited from aqueous suspensions containing ethanol in the range 0-20 vol%.

Table S3. Water contact angle measurements of particles assemblies deposited from aqueous suspensions with various concentration of ethanol.

Symbol of particles	AR	ethanol content in water, vol.%	contact angle
$P(S/PGL)_s3$	AR=6.41	0	53±3
		10	57±2
		20	57±3
		30	57±3
$P(S/PGL)_s4$	AR=8.50	0	56±2
		10	52±1
		20	56±2
		30	54±2

REFERENCES

[1] Fitton, A.O.; Hill, J.; Jane, D.E.; Millar, R. Synthesis of simple oxetanes carrying reactive 2-substituents. Synthesis-Stuttgart **1987**, *12*, 1140–1142.

[2] Namboodiri, V.V.; Varma, R.S. Solvent-free tetrahydropyranylation (THP) of alcohols and phenols and their regeneration by catalytic aluminium chloride hexahydrate. Tetrahedron Lett. **2002**, *43*, 1143–1146.

[3] Halacheva, S.; Rangelov, S.; Tsvetanov, C. Poly(glycidol)-based analogues to Pluronic based copolymers. Synthesis and aqueous solution properties. Macromolecules **2006**, *39*, 6845–6852.

[4] Basinska, T.; Slomkowski, S.; Dworak, A.; Pantchev, I.; Chehimi, M.M. Synthesis and characterization of poly(styrene/α-t-butoxy-ω-vinylbenzyl-polyglycidol) Microspheres. Colloid Polymer Sci. **2001**, *279*, 916-924.

[5] Basinska, T.; Slomkowski, S. Design of polyglycidol-containing microspheres for biomedical applications. Chem. Pap. **2012**, *66*, 352-368.
